# A novel image-based algorithm to support future remote assessment of chewing function

**DOI:** 10.3389/fdmed.2026.1870425

**Published:** 2026-09-03

**Authors:** Dawn Branley-Bell, Richard Brown, Elias Obreque-Sepúlveda, Claire McGrogan, Helen Cartner, Elhassan Mohamed, Chee Siang Ang, Robert Pellegrino

**Affiliations:** 1 School of Psychology, Northumbria University, Newcastle upon Tyne, United Kingdom; 2 School of Engineering, Physics & Mathematics, Northumbria University, Newcastle upon Tyne, United Kingdom; 3 School of Sciences, Psychology, Arts and Humanities, Computing, Engineering, and Sport, Canterbury Christ Church University, Canterbury, Kent, United Kingdom; 4 School of Computing, University of Kent, Canterbury, Kent, United Kingdom; 5 Monell Chemical Senses Center, Philadelphia, PA, United States; 6 Department of Animal Science, Texas A&M University, College Station, TX, United States

**Keywords:** chewing function, comminution, digital health, image analysis, image segmentation, masticatory performance, particle size, remote assessment

## Abstract

**Background:**

Chewing difficulty is associated with poorer physical and mental health. Objective measurement of chewing function is currently limited to methods that require specialist lab equipment (for example lab-based manipulation or comminution tests). A remote method for estimating chewing-related metrics would support the development of accessible and scalable means of detecting and monitoring chewing-related health issues.

**Method:**

This paper details initial work on developing a novel smartphone-based, image-analysis algorithm to estimate particle-size metrics from photographs of masticated raw carrot deposited in a Petri dish. A two-stage image-processing pipeline was developed, comprising geometric calibration and particle segmentation, enabling estimation of particle-size metrics from smartphone photographs. The algorithm was implemented both as a Python script and as a graphical user interface (GUI), the latter allowing semi-automated per-image adjustment of calibration and segmentation parameters with visual feedback. The script and graphical user interface (GUI) are publicly available: osf.io/kgx9f

**Results:**

Performance was evaluated on artificially generated test images to benchmark the algorithm against ground-truth data with known sizes, while also assessing data-collection processes, usability and key challenges. On the more realistic synthetic-particle images, which include overlapping particles of varying size, the algorithm produced a cumulative area error of 20.6% and a mean per-particle relative error of 21.1% (median 14.8%; mean absolute error 1.02 mm^2^), with the largest errors occurring for the smallest and most overlapped particles. These synthetic-particle figures reflect performance on successfully matched particles; end-to-end performance including undetected particles would be lower. On simpler geometric-shape images, total-area relative error was lower, at 3.10% for the large-shape set, 3.00% for the small-shape set, and 3.12% across all shapes.

**Conclusion:**

The algorithm demonstrates quantifiable analytical performance in estimating chewing-related particle-size metrics from smartphone images, supporting its potential use as a foundation for future remote measurement of chewing function. However, the method is not yet clinically validated. In its current form, the method achieves its best performance through a semi-automated, GUI-based workflow, which introduces a subjective element but provides a practical way to handle heterogeneous image conditions and inform future automation.

## 1 Introduction

Chewing is a fundamental aspect of daily life, which plays a crucial role in both physical and mental health. The ability to chew effectively is not only essential for adequate nutrition and safe swallowing but also significantly impacts overall well-being. Chewing difficulty has been linked to various health conditions, including malnutrition, digestive disorders, and psychological distress (1). It is often associated with aging, neurological conditions, and other physical or cognitive impairments (2). Despite its importance, objective measurement of chewing function remains limited and is often restricted to clinical or lab-based settings, where current methods often rely on complex instruments and equipment, or manual evaluations (3). These methods, while useful, are not always accessible for routine monitoring, are resource intensive, and may not reflect daily variability in chewing function.

The need for more accessible, objective, and consistent methods to assess chewing function is clear. This is especially pertinent for individuals with long-term conditions, who may not have regular or adequate access to clinical assessments. The development of a remote, digital system could help address these gaps by supporting future assessment of chewing-related metrics outside lab and clinical settings, without the need for lab visits. This aligns with the trend toward personalised healthcare, which emphasises empowering individuals to monitor and manage their own health.

This paper provides initial work on the development of a novel image-based algorithm designed to support future remote measurement of chewing function by estimating comminution-related particle-size metrics from photographs of chewed food. Using a smartphone camera, this algorithm processes images of chewed food particles to estimate the size and number of particles, which are comminution-related metrics relevant to chewing efficiency. This initial work could inform the future development of broader remote assessment approaches, subject to further validation against established chewing-function measures and testing in independent home settings. This would offer the potential for early detection of changes, allowing for better monitoring of individuals’ health and reducing the need for in-person clinical visits, as well as improving the evaluation of future interventions. The results from this study will inform next steps in testing the feasibility of remote chewing assessment and consider its potential integration into healthcare practices.

### 1.1 Related work

Two established paradigms that attempt to quantify chewing function include: comminution tests, which assess an individual's ability to break down large food items into smaller pieces by analysing the particle-size distribution of chewed test food, and mixing-ability tests, which assess an individual's ability to manipulate food in the mouth by measuring colour mixing in a two-colour test food chewed bolus. Several chewing assessments that incorporate comminution have decades of validation and clinical interpretation (e.g., the Masticatory Normative Indicator based on raw carrot), while mixing tests may offer practicality and ease of implementation (4). Other approaches to assessing chewing function involve batteries of tests measuring bite force, oral tactile sensitivity, and stereognosis, which capture complementary aspects of oral sensorimotor function (5). Additionally, physiological approaches such as electromyography and acoustic analysis have been explored as indirect measures of chewing dynamics and muscle coordination during mastication (6–8).

Most work on comminution is based on manual laboratory techniques, e.g., using wet sieving. However, multiple studies now show that optical scanning (flatbed scanner + image analysis) can produce particle-size distribution outputs comparable to multi-sieve methods, supporting image-based assessment as a valid alternative in standardised settings (9). Additionally, machine learning approaches to estimate particle size are beginning to emerge (10).

An additional method for assessing chewing function involves two-colour gum mixing, which is widely used and has dedicated software and protocols, e.g., ViewGum (11, 12). Recent work has validated smartphone-based applications for the gum test, demonstrating reliability despite some precision loss at higher degrees of mixing (12). Although both mixing and comminution arise from the same masticatory processes, mixing tests primarily quantify bolus homogenisation and coordination, whereas comminution-based measures explicitly characterise particle-size reduction, a central outcome in the clinical interpretation of chewing efficiency.

Building on this related work, our approach targets remote, low-burden comminution assessment by: (1) using smartphone photographs of chewed raw carrot (a standard test food) on a standardised mat; (2) implementing an assessment of standardised images using a rigorously developed algorithm.

This seeks to extend image-based comminution methods toward potential home-capture settings, complementing the adoption of smartphone technology recently tested in mixing-ability workflows, but directly addressing particle breakdown.

## 2 Materials and methods

Here we set out the decisions and processes involved in: (1) Image capture, including the choice of image target, parameters, and standardisation of capture conditions; (2) Algorithm development, including calibration and determination; and (3) Algorithm performance evaluation, including testing with simple and complex synthetic images (to benchmark against ground truth data with known sizes), parameter sensitivity, and implementation.

The present manuscript focuses on algorithm development and analytical validation using generated geometric shapes and synthetic particle images with known ground-truth values. Participant-based feasibility and usability work informed the development of the image-capture protocol but is not analysed as part of the present manuscript and is reported separately (see https://osf.io/kgx9f).

### 2.1 Image capture

To ensure that image analysis accurately reflected chewing function, particular attention was given to sample preparation and image capture. Several rounds of testing were undertaken to establish the most effective methods for producing consistent, high-quality images suitable for automated analysis. This is of particular importance given that imaging conditions need to reflect what individuals could realistically be expected to achieve independently in a non-lab setting.

#### 2.1.1 Choice of food model: carrot

The selection of image type was a key early consideration in enabling an automated and remote assessment approach. Based on prior literature, and considering accessibility and convenience, we selected images of masticated raw carrot particles deposited in a square Petri dish as the basis for chewing assessment. These were photographed using a standardised collection process. Carrot was chosen for several reasons: it is commonly used in chewing efficiency tasks (4, 13); it is inexpensive and widely available; it can be cut to a standard size and shape; and it avoids potential issues with common allergies, such as nuts (4). Carrot is brightly coloured, which facilitates the use of colour thresholds in algorithm development. Finally, as a ‘real’ as opposed to artificial test food, carrot is arguably more ecologically valid (14), generating a more natural chewing action (15, 16).

#### 2.1.2 Selected image parameters

Image parameters including particle count and particle size (minimum, maximum, mean, and median) were selected as proxies for chewing function based on the mastication literature (4, 17, 18). In comminution tests, performance is typically assessed by the median particle size. We also report mean size as a complementary central tendency, and minimum and maximum as descriptive checks for outliers such as unusually large fragments suggesting incomplete comminution (17). Particle count was included as it typically rises with smaller particle sizes and has precedent in related sieve-based measures of masticatory performance (18). Finally, these parameters were chosen for their clinical relevance: lower performance on these measures reflects reduced masticatory function, which is linked to impaired oral health and broader health outcomes (19).

#### 2.1.3 Sample preparation for image capture

To ensure that image analysis accurately reflected chewing function, preparation of the masticated carrot sample before imaging was a critical consideration. Key factors included the dimensions of the original carrot piece prior to chewing and the number of chews before depositing in the Petri dish (in order to balance realistic effort with sufficient breakdown for analysis); the amount of water rinse immediately before spitting (to disperse particles evenly and reduce clumping); and manual separation of particles prior to photographing (to assess whether separation improved measurement accuracy). These variables were evaluated to establish the most consistent and representative method for preparing chewed carrot samples.

#### 2.1.4 Optimal image capture conditions

Reliable generation of acceptable-quality images required a standardised capture process. To establish optimal conditions, we developed a printed mat to sit beneath the Petri dish, incorporating a black reference dot (20 mm diameter) for algorithm calibration of scale and alignment. A plastic light box was then adopted to standardise lighting, shadow effects, camera distance and angle during photography (see Additional File A1). A photo distance of 20 cm was selected to ensure full capture while maintaining image quality. Testing of background colours confirmed that a white background provided the clearest definition.

### 2.2 Algorithm development

**Figure 1:**
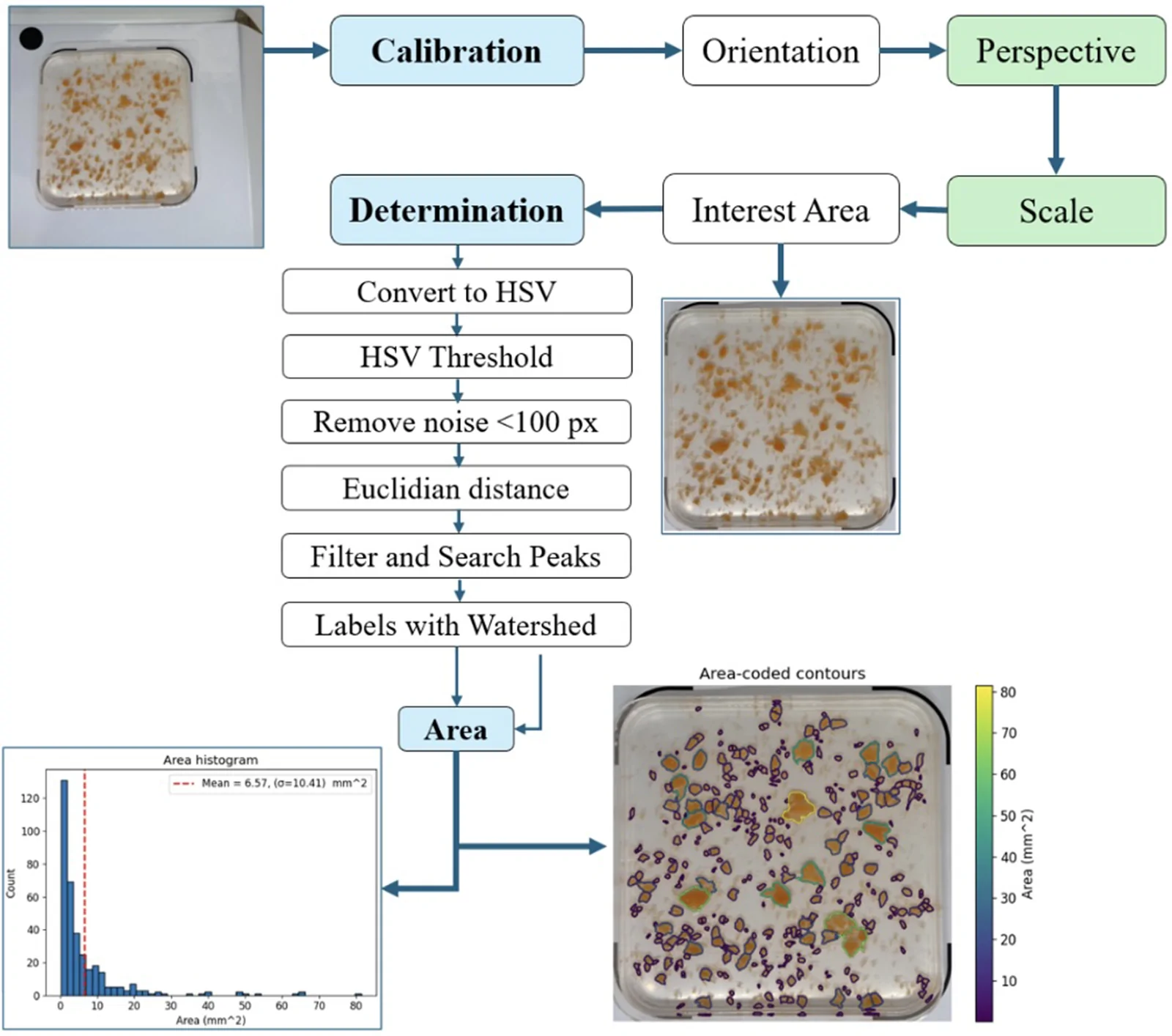
Diagram of workflow for algorithm development: calibration and determination.

Algorithm development was initially undertaken in MATLAB, a widely used platform for image-based algorithm prototyping (17), before being converted into Python to enable open-source implementation and integration with established image-processing libraries (e.g., OpenCV, scikit-image, NumPy) (20). The aim was to automate analysis of selected images and generate the target image parameters. The workflow comprised two main stages. In the calibration process, each image was standardised for orientation, perspective, and scale. In the determination process, food particles were isolated by colour, separated when touching, and overlaid with simple geometric outlines to ensure consistency in size estimation. The resulting pipeline is deterministic and implemented using widely adopted open-source tools in Python. An overview of the workflow is presented in Figure 1 (see also Additional File A2 for a more detailed breakdown of the workflow stages for algorithm development). Initial evaluation of algorithm performance used a series of printed test images containing geometric shapes of varying known sizes to simulate particle distributions. Further evaluation involved generating an artificial image composed of small ellipses to more closely approximate the size and shape characteristics of chewed carrot particles.

#### 2.2.1 Calibration

The following calibration process standardises each image by correcting orientation, rectifying perspective to a front-on view, and defining a pixel-to-millimetre scale using the printed reference circle:

a) Orientation. Photos are loaded and automatically rotated according to the camera’s EXIF (Exchangeable Image File Format) orientation tag, ensuring all images start upright. This reduces variability due to how the camera was held.
b) Perspective. To correct for viewing-angle distortion, the rectangular container in the image is detected and the perspective adjusted. The process starts by converting the image from RGB to a LAB colour space—which splits colours into three separate channels based on L channel (Lightness), A channel (green-magenta axis) and B channel (blue-yellow axis). Images are typically captured in RGB (red, green, and blue) colour, but this makes detecting black difficult. Since reference corners are black, the brightness (L) spectrum of the LAB colour helps to detect them. The image was then eroded to remove text and dilated to strengthen lines. This step uses edges and straight-line detection to locate all the lines. Then, four average lines are computed for each side applying a perspective transform to the full image. A cropped (Interest Area), rectified view around the container is also produced for subsequent analysis.
c) Scale. The black reference circle situated in the corner of the printed mat is automatically located in the rectified image using Otsu Threshold and Ellipse fit. Its measured size in pixels defines the pixel-to-millimetre conversion used for all area computations (pixels per millimetre). This removes differences due to camera lens distance, focal length or zoom. To identify the reference circle and assess its quality, two metrics are used: circularity and darkness score.

The circularity score is defined as:

$$C=4\;\pi \cdot \frac{Area}{Perimeter^{2}}$$

The darkness score is defined as:

$$Score=1-\;\frac{I_{m}}{255}$$

Where $I_{m}$ is the mean colour value of the candidate circle pixels contour.

#### 2.2.2 Determination

The following determination process segments food fragments, removes artefacts, separates touching pieces, and computes their areas in mm^2^.

a) Colour segmentation. The rectified image is converted from the original colour RGB to HSV colour space, and a fixed HSV range is applied to isolate the target food tones (orange/brown). This produces a binary mask in which candidate food pixels are set to 1 and the background to 0.
b) Noise cleaning. To prevent reflections and non-food detritus from affecting the size distribution, small specks and artefacts were removed by eliminating connected components below a fixed physical size of 0.57 mm^2^ (a circle of ∼0.85 mm diameter). This cut-off lies well below the particle sizes relevant to comminution assessment (typically a few mm), so it removes noise without discarding genuine fragments. The threshold is defined in physical units and recomputed from the calibrated scale of each image: at the working resolution of 9.4 px/mm it corresponds to ∼50 px, and it rescales automatically when the resolution changes, preserving the same physical cut-off.
c) Particle Separation. Because particles can touch or slightly overlap in the image, we computed a Euclidean distance transform on the cleaned mask and identified local peaks as tentative centres of individual pieces. These peaks were used as markers for a watershed transform, which split the mask into distinct labelled regions, one label per fragment. The watershed method attempts to solve the problem of superimposing sample geometry, although it is noted that it is sensitive to particle shadows, colours, and shapes.
d) Area computation (mm^2^). The final area reported for each particle is the mathematical area of a fitted ellipse (π × semi-major axis×semi-minor axis), rather than a raw pixel count. The algorithm employs a two-step fitting process: initially, an ellipse is fitted to the boundary contour of the watershed-segmented region. If this contour-based fit yields an Intersection-over-Union (IoU) below 0.65, an “ellipse adjustment” step is triggered, which recalculates the ellipse axes using the spatial pixel moments of the region rather than its edges, giving a more stable fit for highly irregular particles. In both cases the reported area is that of the resulting ellipse. Areas were converted to physical units using the image-specific pixel-to-millimetre factor obtained during calibration, yielding size determination independent of camera lens distance, focal range or zoom.
e) Quality control and visualisation. To facilitate review, we overlaid contours of each labelled region on the original image and colour-coded the outlines by area. We also summarised the distribution with a histogram and reported the mean (and standard deviation).

## 3 Results

The algorithm was iteratively refined and tested for accuracy in calculating relevant image metrics. Performance was evaluated using two primary measures: particle count and particle size estimates.

### 3.1 Evaluation with simple geometric shapes

We first evaluated the algorithm on simple geometric shapes with known areas, to establish baseline accuracy under controlled, well-defined conditions. We evaluated on artificially generated images with known sizes due to the lack of ground truth datasets for real food particle images.

**Figure 2:**
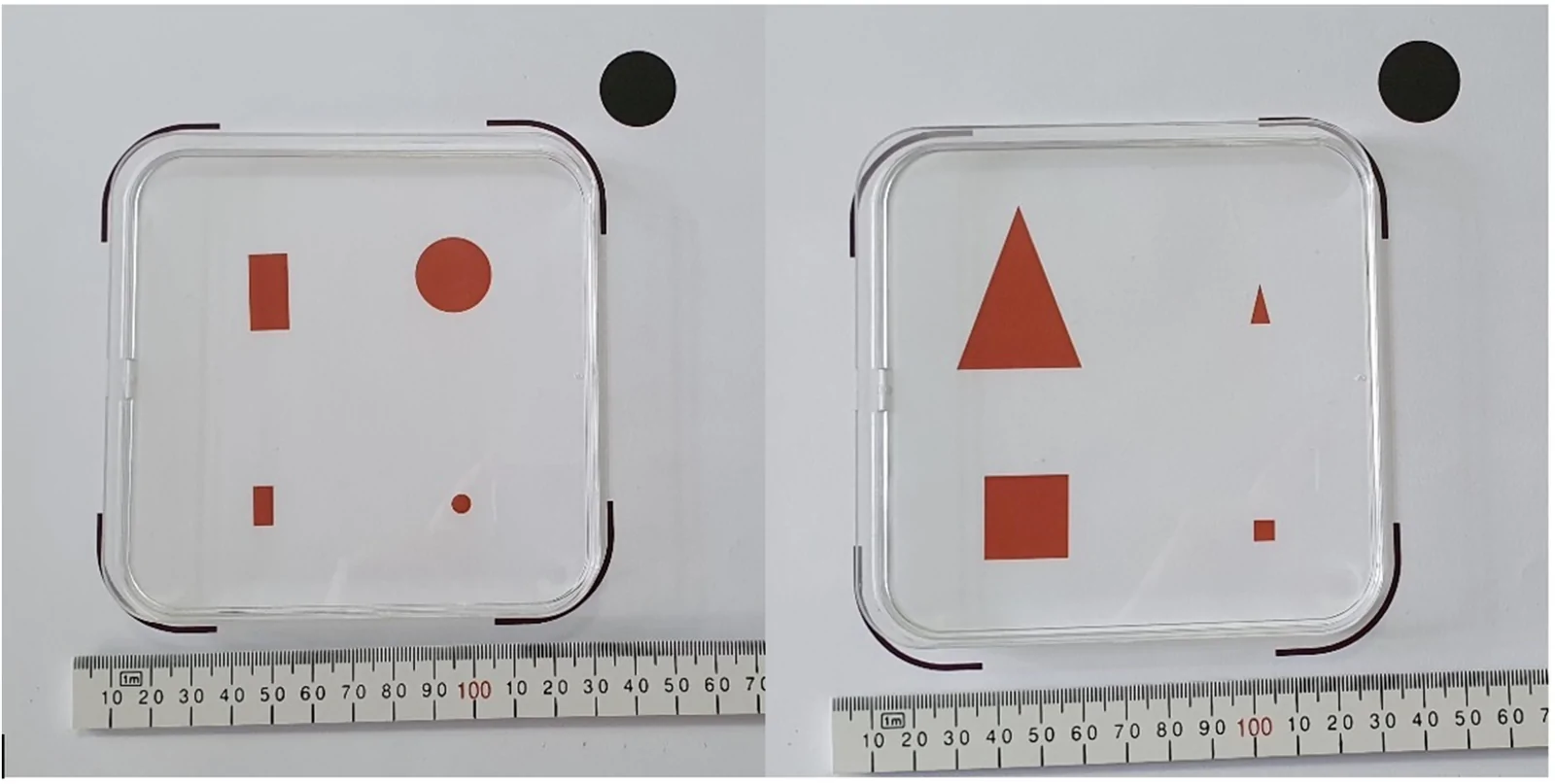
Generated test images for evaluating algorithm performance on standard geometric shapes.

Particle count estimates were derived from the labels obtained via watershed segmentation (see Section 3.2). This step attempts to ensure that the fragments in contact are separated and counted individually, allowing a reliable estimate of the count regardless of the particle distribution, whether very narrow or very wide. Particle size estimates were evaluated using artificially generated images containing four standard geometric shapes of known areas (see Figure 2). The shapes were designed to replicate the approximate colour of the target test food (carrot). The algorithmically calculated area values for the shapes were then compared against the known true values to assess performance.

The overall percentage error for the estimated total area of the geometric shapes was 3.12% (see Table 1), calculated using the relative error $M=\left(\frac{estimate}{actual}-1\right)100\;\text{\%}$. In this formulation a positive value indicates that the algorithm overestimated the true area and a negative value indicates an underestimate, so the sign denotes the direction of the error; total-area figures in the text are reported as magnitudes. This overall value combines the large- and small-shape test sets. When analysed separately, the total-area relative error was 3.10% for the large-shape set and 3.00% for the small-shape set. Table 1 reports areas in cm^2^ because the validation shapes were designed and measured at centimetre scale; the later synthetic-particle analysis reports areas in mm^2^ because the ellipses approximate smaller chewed-particle fragments. Individual shape-level errors varied, with the largest magnitude observed for the large circle (6.37%). These differences likely reflect boundary-thresholding effects, as the algorithm must decide whether edge pixels belong to the shape or the background (which may be worsened when shapes have a curved edge due to more ambiguous pixels). Notably, the algorithm underestimated the area of every geometric shape (except the small square, which was measured precisely), indicating a small systematic tendency to under-segment rather than random error in both directions, consistent with a conservative edge-thresholding effect. However, total-area errors were similar across the large and small test sets.

**Table 1:** Algorithm evaluation for particle size area estimates for multiple geometric shapes.

Standardised Shapes	Actual area of shape (cm^2^)	Algorithm Estimate (cm^2^)	Relative Error (%)
Large Triangle	6.00	5.91	−1.50
Large Square	4.00	3.92	−2.00
Large Circle	3.14	2.94	−6.37
Large Rectangle	2.00	1.90	−5.00
Small Circle	0.20	0.19	−3.06
Small Rectangle	0.50	0.48	−4.00
Small Triangle	0.25	0.24	−4.00
Small Square	0.25	0.25	0.00
Total for large	15.14	14.67	−3.10
Total for small	1.20	1.16	−3.00
Total	16.34	15.83	−3.12

Percentages calculated from unrounded area values; recomputing from the rounded figures shown may differ slightly.

### 3.2 Evaluation with complex synthetic images

**Figure 3:**
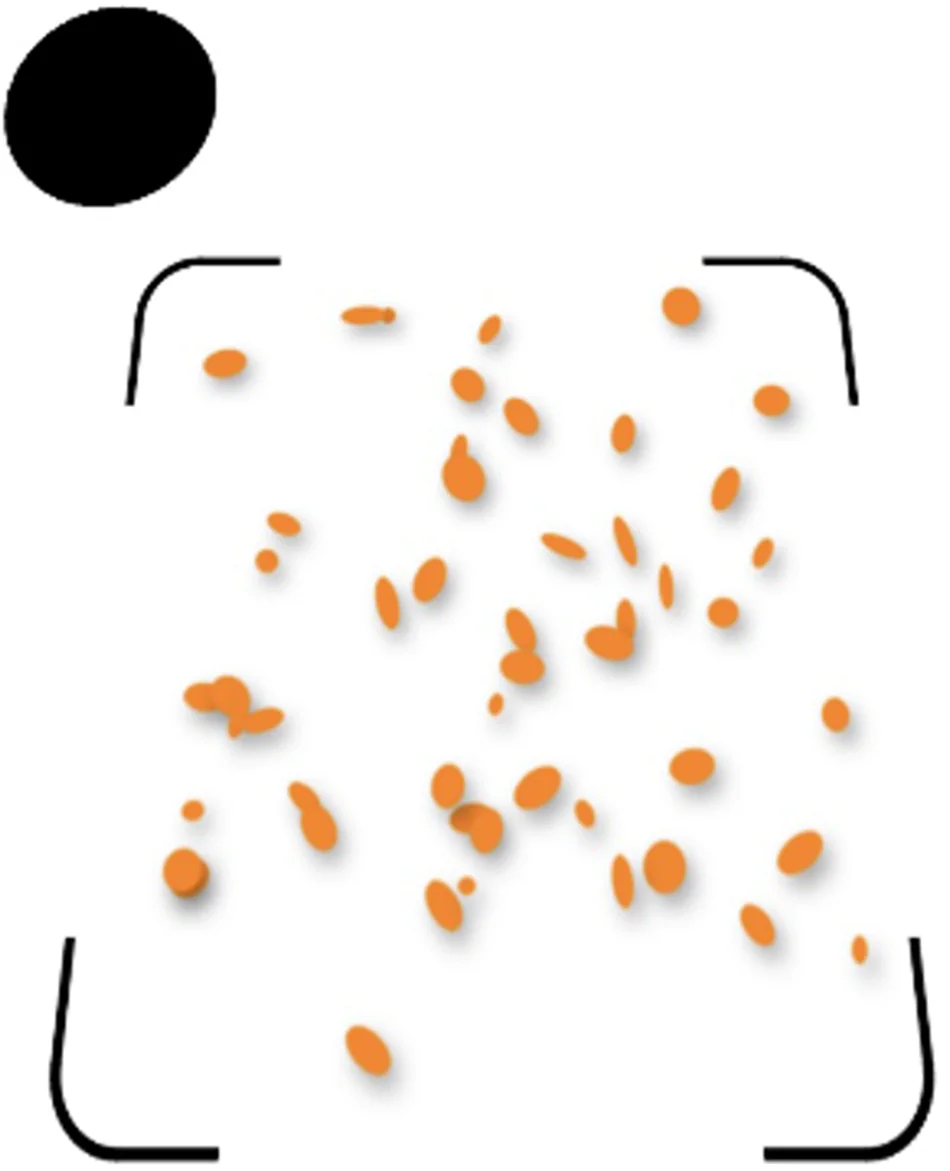
Synthetic image with known dimensions and projection.

**Figure 4:**
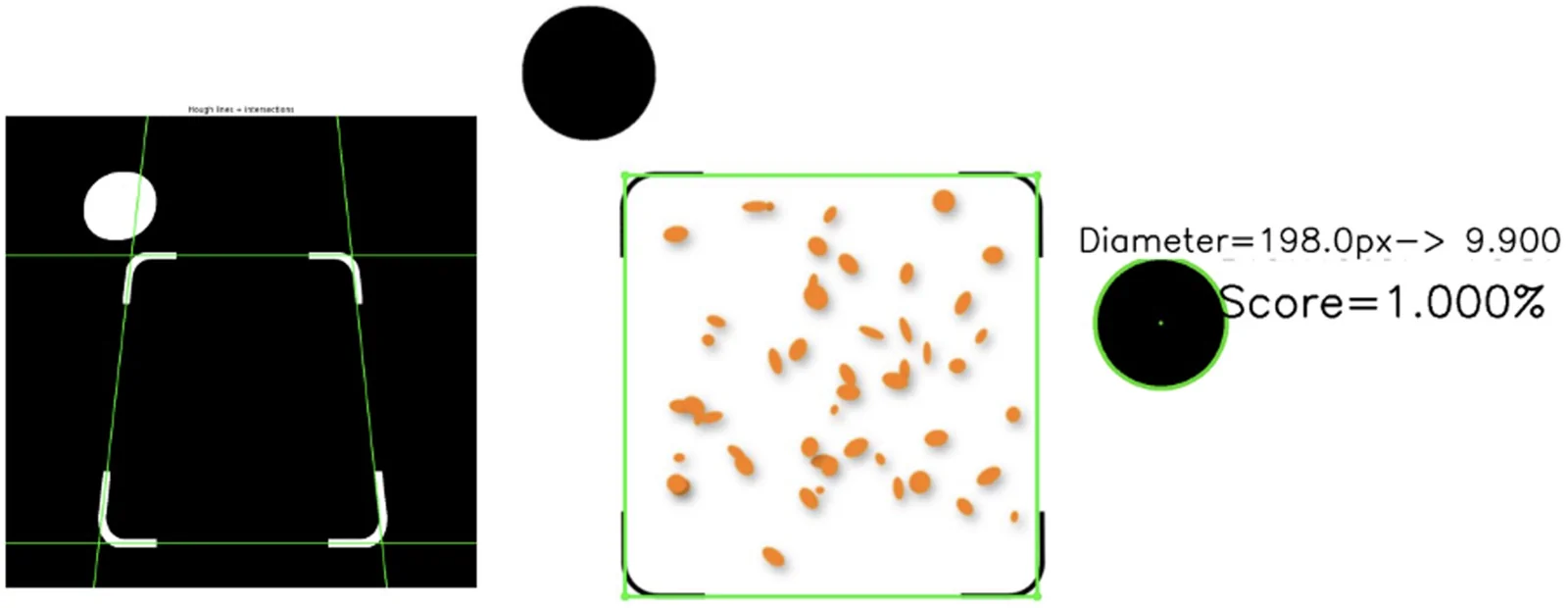
Processing the synthetic image.

To further evaluate algorithm performance in capturing small particle size, we created an artificial image of small ellipses (again the same colour as carrot, Figure 3). This step was included because in comparison to the previous shapes, the ellipses provide a closer approximation to key properties of chewed carrot while retaining full ground truth. Ellipses capture the smaller, more frequent, rounded, and often overlapping, fragments seen after comminution, and can be generated across a realistic size range with controlled spacing and overlap. This image includes samples of 50 ellipses of random and known sizes and positions, the scale reference circle, and a known tilted projection of 6.4°. The synthetic image is shown in Figure 3. The objective of this evaluation is to recalculate the area of each ellipse and compare it with the original value. Figure 4 shows the results of the calibration process, which, after projection, determines the dimensions of the Reference Scale (Diamete*r* = 198 px).

**Figure 5:**
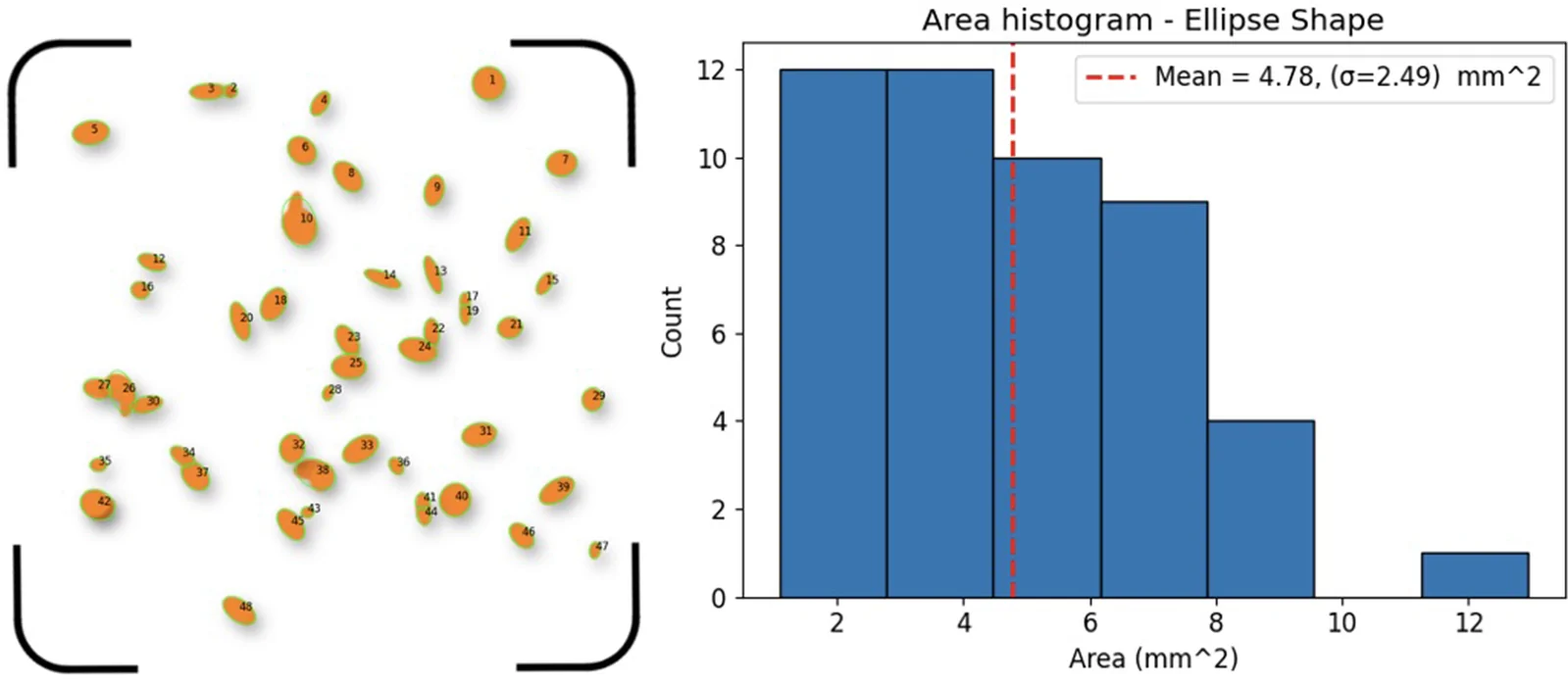
Area detected with ellipse adjustment. The histogram shows the distribution of the detected areas and their standard deviation (95 percentile).

**Figure 6:**
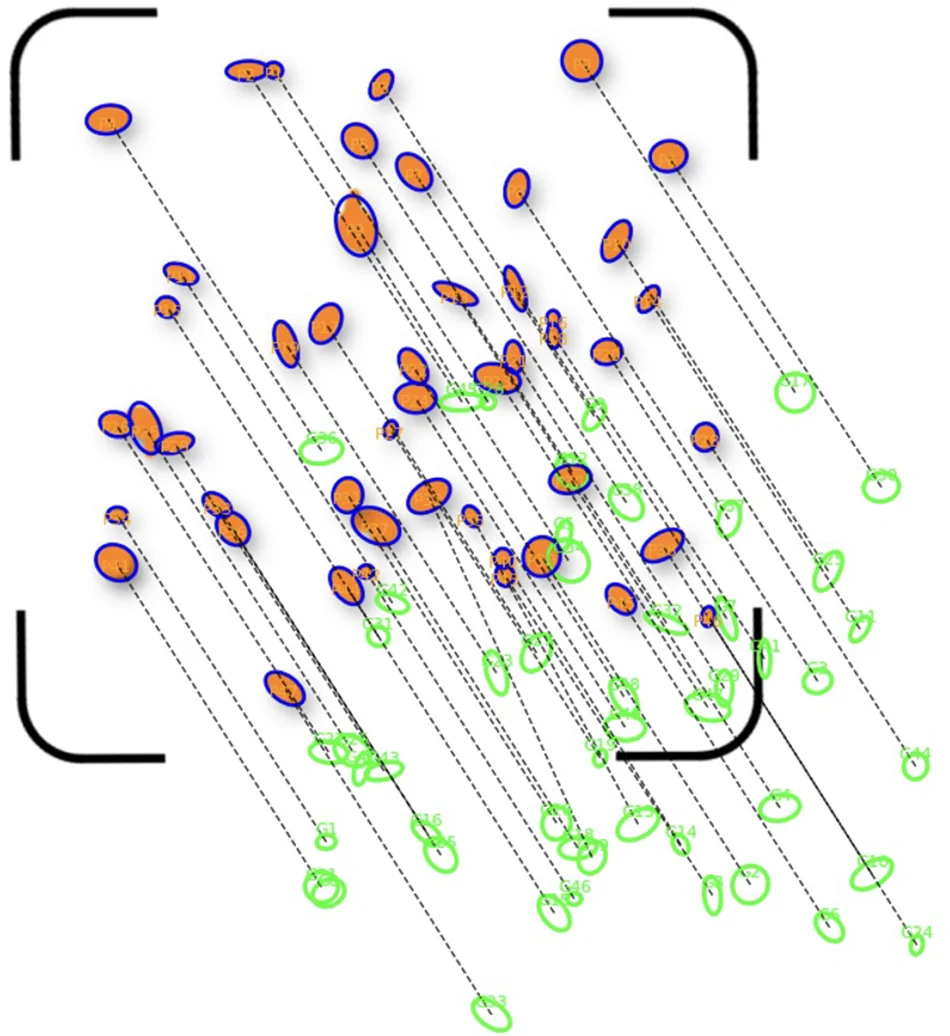
Matching the known ellipses (ground truth with green colour) with the calculated ellipses (blue).

The algorithm calculates the area of each segmented sample by the watershed method. These results are shown in Figure 5 and Table 2. Detected ellipses were matched to the ground-truth ellipses by point-set registration followed by optimal assignment. Because the ground-truth coordinates are defined in the synthetic-image frame prior to the simulated perspective projection, whereas detections are measured in the rectified, cropped frame, a 2D similarity transform (scale, rotation, translation) was first estimated between the two-point sets using a RANSAC (Random Sample Consensus) scheme on scale- and translation-invariant local neighbourhood signatures. Detections were then transformed into the ground-truth frame and assigned by the Hungarian algorithm minimising centroid distance, subject to a distance gate. As shown in Figure 6, the algorithm first detected 47 of the 50 ground-truth ellipses (a particle-count error of −6%); the 3 undetected ellipses lay in regions of severe overlap that were not resolved into distinct labels. Of the 47 detections, 45 were successfully matched to their ground-truth counterparts, while 2 fell outside the distance gate and were left unmatched. Matching therefore yielded a recall of 0.90, a precision of 0.96, and an F1 score of 0.93. Recall is calculated relative to the 50 ground-truth ellipses, whereas precision is calculated relative to the 47 detected ellipses. The 5 ellipses that were either undetected or unmatched were excluded from the area-error statistics rather than assigned a zero or maximum error. The reference scale was recovered accurately, with a calibration error of −1.0%. To characterise agreement between estimated and true areas, a Bland-Altman analysis of the 45 matched ellipses indicated a small systematic overestimation, with a mean bias of +0.93 mm^2^ (computed as estimated minus true area, so a positive value denotes overestimation) and 95% limits of agreement of [−1.28, 3.14] mm^2^. Relative error was largest for the smallest particles, consistent with the boundary- and separation-sensitivity described above.

**Table 2:** Error in calculated areas for paired ellipses in the synthetic image.

Match	Ground truth Area (mm^2^)	Determined Area (mm^2^)	Absolute Error (mm^2^)	Relative Error (%)
1	5.64	9.91	4.26	75.59
2	9.74	13.84	4.10	42.05
3	4.94	8.85	3.91	79.30
4	4.94	8.28	3.35	67.81
5	3.59	6.57	2.98	82.87
6	5.39	7.31	1.93	35.77
7	2.56	0.75	1.81	−70.57
8	6.15	7.49	1.33	21.63
9	1.15	2.45	1.29	111.99
10	5.99	7.07	1.08	18.00
11	8.21	9.17	0.96	11.75
12	6.35	7.19	0.85	13.35
13	7.69	8.50	0.80	10.46
14	6.09	6.89	0.80	13.10
15	5.77	6.56	0.79	13.66
16	5.99	6.77	0.78	13.02
17	5.99	6.77	0.78	12.96
18	3.65	4.43	0.77	21.18
19	6.70	7.47	0.77	11.44
20	5.19	5.96	0.76	14.70
21	5.99	6.76	0.76	12.71
22	4.87	5.63	0.76	15.51
23	3.65	4.41	0.75	20.59
24	5.77	6.50	0.73	12.64
25	5.77	6.47	0.70	12.11
26	6.70	7.39	0.69	10.30
27	5.45	6.12	0.67	12.28
28	4.33	4.99	0.66	15.30
29	3.14	3.71	0.57	18.05
30	2.92	3.47	0.55	19.01
31	3.59	4.14	0.55	15.39
32	3.85	4.39	0.54	14.14
33	3.53	4.05	0.52	14.81
34	4.04	4.55	0.51	12.61
35	2.31	2.76	0.45	19.63
36	1.73	2.07	0.34	19.52
37	4.90	5.23	0.32	6.60
38	2.31	2.61	0.31	13.23
39	1.54	1.82	0.28	18.29
40	1.12	1.40	0.28	24.62
41	1.28	1.52	0.24	18.84
42	0.96	1.17	0.21	21.82
43	3.37	3.55	0.18	5.37
44	4.26	4.14	0.12	−2.85
45	4.62	4.63	0.01	0.29
Total	203.73	245.69	41.95	20.6

Relative error is calculated as M = (estimate/actual−1) × 100%, where a positive value indicates that the algorithm overestimated the true area and a negative value indicates an underestimate. Values are shown for the 45 matched ellipses only; the 5 unmatched ellipses (3 undetected and 2 falling outside the distance gate) were excluded from the area-error statistics rather than assigned a zero or maximum error.

The area errors for matched ellipses are reported in Table 2, with a cumulative area error of 20.6% and a median relative error of 14.8% per particle (mean 21.1%). The mean absolute error is 1.02 mm^2^ with a standard deviation of 1.04 mm^2^. Two of the largest deviations illustrate both directions of this watershed-separation effect: merged overlapping ellipses produce inflated areas (e.g., an estimate of 2.45 mm^2^ against a true 1.15 mm^2^, ≈2×), while over-separation produces underestimates (e.g., 0.75 mm^2^ against a true 2.56 mm^2^, −71%). These statistics are calculated for the 45 successfully matched particles only and therefore describe segmentation and area-estimation accuracy where a particle was both detected and matched. They do not capture end-to-end performance, which would additionally be affected by undetected and unmatched particles; the effect of those particles is reported separately as detection and matching accuracy.

**Figure 7:**
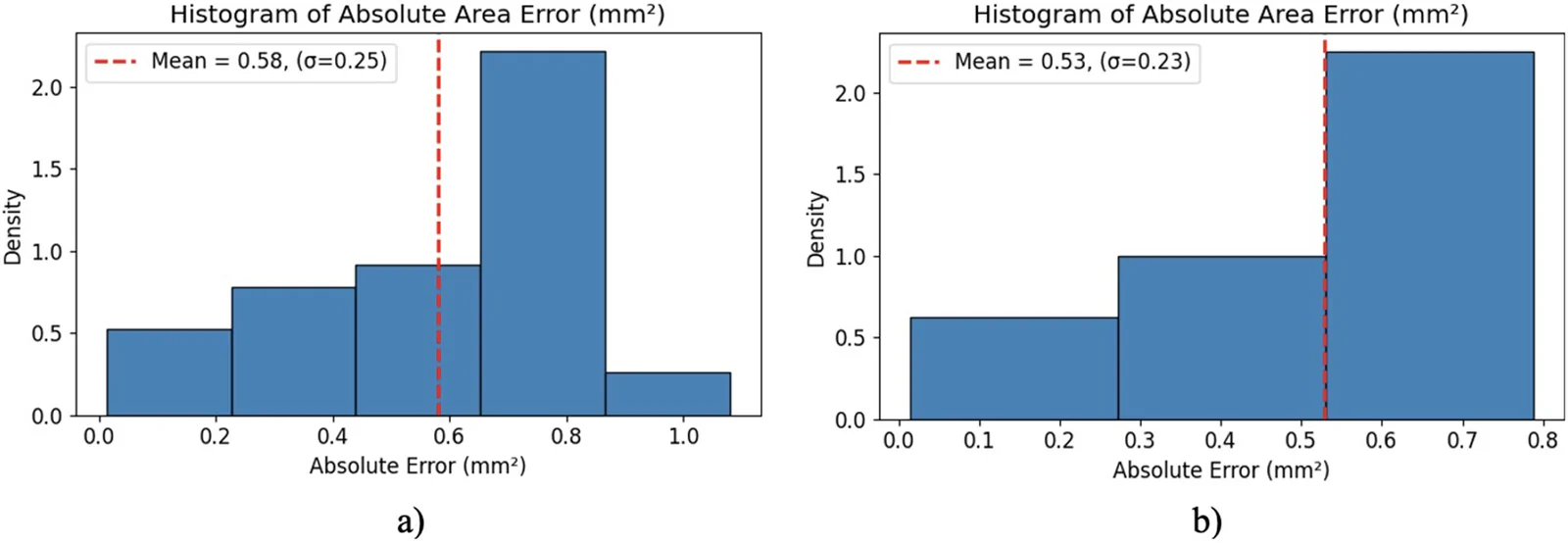
Absolute error of the ellipses matched*.* **a**) Using the 80% percentile, the outliers are mainly due to the ellipses that failed to match. **b**) Using 70% percentile, the outliers were removed to calculate the mean and standard deviation.

Since particle overlap was included in this test, this influenced the mean and standard deviation of the area estimates. As an exploratory sensitivity analysis, we also examined error distributions after excluding the largest errors using percentile thresholds. These percentile-filtered results are not presented as the primary performance estimate, as overlapping particles and segmentation outliers are likely to occur in real masticated samples. Instead, they illustrate the extent to which a small number of poorly estimated particles contributed to the overall error distribution. Figure 7 shows the distribution of the estimation error for different percentile levels, 80% and 70%, reducing the absolute error from 1.02 mm^2^ to 0.58 mm^2^ and 0.53 mm^2^, respectively. The larger effect, however, is on the standard deviation, going down from 1.04 mm^2^ to 0.25 mm^2^ (80%) and 0.23 mm^2^ (70%) respectively.

### 3.3 Algorithm development summary

#### 3.3.1 Evaluation overview

Across the geometric-shape and synthetic-ellipses tests, the algorithm achieved low errors on simple isolated shapes but larger errors under the more realistic conditions of overlapping particles, tilt and variable lighting. No predefined threshold for acceptable accuracy was set; performance is therefore reported descriptively rather than judged against a fixed criterion. As a general evaluation, the synthetic-ellipses test produced a cumulative area error of 20.6% and a mean per-particle relative error of 21.1% (minimum below 1%). The full-error results are retained as the primary estimate of algorithm performance. However, exploratory percentile-filtered sensitivity analyses indicated that a small number of poorly estimated or highly overlapping particles contributed disproportionately to the overall error distribution. When considering the 80th percentile, the absolute error reduced from 1.02 mm^2^ to 0.58 mm^2^ and the standard deviation reduced from 1.04 mm^2^ to 0.25 mm^2^. This constitutes an initial proof-of-concept evaluation of a remote comminution algorithm pipeline that is potentially suitable for remote-capture images.

#### 3.3.2 Parameter sensitivity and semi-automated implementation

Extensive efforts were made to standardise image capture, including the use of a lightbox, fixed camera distances, grid markings and reference points. Nevertheless, these image capture conditions still resulted in inconsistent conditions between photographs, for example differences in lighting, reflections from the Petri dish, partial occlusion of grid markers and variable visibility of the reference circle. These factors introduce noise into the images and influence both the calibration step and the segmentation of particles. Manual tuning of GUI parameters was undertaken by a project-team data scientist with expertise in image capture and image analysis. Acceptable calibration and segmentation were judged through visual inspection, based on whether the selected region of interest aligned with the target image area and whether the resulting particle labels provided a reasonable representation of the visible carrot fragments.

Performance remains sensitive to the configuration of calibration and determination parameters. For example, the algorithm can favour larger or smaller particles depending on the selected filters and thresholds, such as saturation and brightness levels, and the minimum distance between segments used in the watershed method. Configuration parameters are therefore key to correctly estimating particle areas, and their optimal values depend on the characteristics of each individual image. The default values of these configuration parameters are summarised in Table 3. In practice, this parameter dependence means that the choice of settings for both calibration (erosion, dilation, line gaps and reference scale) and area determination (saturation and brightness cut-offs and minimum sample distance) has a notable influence on the algorithm's outputs. Given the variability in image conditions, the best results at present are obtained using a semi-automated approach in which a user visually inspects the calibration and segmentation outputs and adjusts parameters to achieve a satisfactory representation of the chewed particles.

**Table 3:** Internal algorithm parameters (default script configuration).

Stage	Parameter	Value
Perspective correction	LAB mask: lightness threshold (L)	<120
	LAB mask: chroma threshold (C)	<20
	Morphological close (kernel)	5 × 5, 1 iteration
	Erosion/dilation (3 × 3 kernel)	1/4 iterations
	Canny hysteresis (low/high)	60/100
	Edge ring mask (band)	0.55
	Hough threshold	25
	Hough min line length	0.1 × image height
	Hough max line gap	0.5 × image height
	Line orientation tolerance	vertical 75–105°; horizontal <15°/ >165°
Scale/reference circle	Gaussian blur (kernel, *σ*)	(7,7), *σ* = 1.6
	Threshold	Otsu (binary inverse)
	Morphological open/erode	50 × 50 ellipse/4 × 4, 1 iteration each
	Min contour area	300 px
	Min circularity	0.85
	Radius search range	[0.01, 0.28] × min(H, W)
	Reference diameter	20 mm
	Scale factor	2r/20 mm
Colour segmentation	HSV lower bound (H, S, V)	[0, 80, 100]
	HSV upper bound (H, S, V)	[255, 255, 255]
Noise removal	Min object size	0.57 mm^2^ (∼0.85 mm Ø)
Watershed	Min distance between peaks	8 px
	Pre-erosion (disk radius)	5
	Compactness	0.02
Ellipse fitting	IoU acceptance threshold	0.65
	Contour smoothing	1

To support this, two implementations of the code are provided. The first is a script-based version that runs the full pipeline in Python with a set of default parameters. The second is a graphical user interface (GUI) that exposes the same key parameters via sliders and checkboxes and provides immediate visual feedback on calibration and particle segmentation—this allows experienced users to manipulate parameters if needed. This semi-automated GUI workflow introduces an inherently subjective element, since users decide which segmentation best reflects the chewed carrot particles, but it currently offers the most consistent results across heterogeneous images, albeit without a predefined performance criterion. At the same time, it provides an important initial proof of concept for algorithmically estimating particle counts and areas from digital images of chewed food. This semi-automated approach may also offer a foundation for future development toward adaptive or fully automated methods.

Further details of the semi-automated workflow and practical guidance for parameter adjustment are provided in the accompanying user guide to the image-based chewing estimation algorithm.

#### 3.3.3 Availability of data, code and graphical user interface (GUI)

Annotated scripts and sample images are available on the Open Science Framework (OSF; https://osf.io/kgx9f). The GUI, user guide, and README file with step-by-step setup instructions are included to support further testing and development. The GUI enables non-expert users to: (i) load images, (ii) adjust calibration and area-detection parameters, (iii) visually inspect segmentation and area estimates, and (iv) export relevant metrics. Default presets are provided, calibrated on the sample smartphone images of masticated carrot, with manual tuning available for atypical lighting or projection conditions. Together, these resources are intended to make the current prototype reproducible and practically adoptable for further testing and development. The combination of a script-based implementation and a semi-automated GUI lays the groundwork for future stages of work, including improved image capture procedures, refinement of the algorithm's adaptability, and the development of more fully automated parameter selection and large-scale validation.

## 4 Discussion

This study presents a proof-of-concept image-based algorithm for estimating comminution metrics from photographs of chewed carrot samples. The approach demonstrates that particle count and particle-size summaries can be estimated from smartphone images under controlled conditions, providing an analytical foundation for future work on remote measurement of chewing function. This work bridges validated optical comminution methods and real-world home capture by combining a standardised mat, scale reference, and a deterministic two-stage algorithmic pipeline (calibration → determination), demonstrating that interpretable comminution metrics (chewed particle count and size) can be derived from smartphone images under non-lab conditions. In practice, the best performance at present is achieved using a semi-automated implementation, in which users adjust calibration and segmentation parameters via a GUI and rely on visual inspection to judge acceptable outputs. This introduces a subjective element into the workflow but provides a practical way to handle heterogeneous image conditions and an initial foundation for developing more adaptive, fully automated versions of the algorithm. The following discussion considers the opportunities and challenges of remote assessment, the clinical interpretability of the outputs, and key technical considerations and directions for future work.

### 4.1 Remote assessment: opportunities and challenges

In the longer term, remote, low-burden monitoring of chewing function may be feasible if image-based particle-size metrics can be validated against established chewing-function measures and collected reliably in home settings. Such an approach could eventually offer a way to track changes between appointments without requiring routine clinic or lab visits for chewing assessment. By bringing measurement into everyday contexts, it can complement lab or clinical tests with richer longitudinal data and greater convenience, consistent with broader trends in remote patient monitoring and home-based digital health (21, 22). However, development of this system has been shaped throughout by the need to balance optimal image-capture conditions against the practical constraints of remote use—the equipment complexity, assembly and operation that individuals can realistically manage independently at home.

Although participant-based feasibility and Think Aloud work informed the practical development of the image-capture protocol, those data are not analysed in the present manuscript. They are described here to contextualise the practical challenges that future remote implementation will need to address. Initial feasibility trials of image capture of chewed carrot particles by participants were lab-based to allow supervision and support where necessary, and involved equipment (light box, printed mat with black reference dot, same smartphone—iPhone 11, iOS 18.5) assembled and positioned by researchers. Participants were also healthy individuals with no self-reported chewing difficulty. While this trial was an essential early stage in assessing optical capture and measurement of chewing function, it is of questionable ecological validity and utility in assessing fully independent use of the system. Subsequently, ‘Think Aloud’ methodology (23) was applied in a further trial in which participants were presented with a bag of equipment and written step by step instructions (illustrated with photos) and asked to assemble the lightbox, prepare standardised carrot pieces for the chewing tasks and position the printed mat and smartphone to photograph chewed carrot particles, voicing their thoughts as they did so. Findings from this study (https://osf.io/kgx9f/overview) offered considerable insight into potential issues which could impact independent use of the system, optical capture of chewing task outputs (carrot particles) and application and evaluation of the algorithm in less controlled or standardised conditions. Successful use depends on people being able to use the supplied equipment and follow the instructions, which may be harder for those with lower agency due to illness, age, limited mobility, or reduced fine motor control. Effective remote assessments of chewing ability will depend on practical safeguards, co-designed guidance, capture-quality prompts, minimal set-up requirements, and clear options for caregiver assistance.

Even when a standardised grid, fixed scale reference, and supplied lightbox, real-world home environments will introduce variability. Image conditions will vary with lighting, shadows, surface reflections, device camera quality and angle, and these sources of heterogeneity cannot be eliminated by protocol alone, particularly in older or more vulnerable populations who are already the focus of much teledentistry work (24, 25). As a result, future versions of the algorithm must prioritise robustness to inconsistent capture conditions, including the ability to adapt thresholds dynamically to noise, rather than relying solely on stricter instructions.

### 4.2 Clinical interpretability

Our findings address analytical validity by examining whether the algorithm measures area and count under controlled conditions, but not yet clinical validity (agreement with reference tests in patients) or clinical utility (impact on care decisions and patient wellbeing). Ultimately, the value of remote assessment lies in producing outputs that inform patients and clinicians and can be related to existing tests. This initial work offers an algorithm that reports particle-size summaries and particle count. These measures are consistent with the comminution and masticatory-assessment literature, which typically emphasises particle-size distributions and count as indicators of masticatory performance (4, 17). Lower performance usually corresponds to larger fragments and/or fewer particles, but interpretation must be anchored to established reference thresholds.

In practical terms, median particle size would likely provide the primary comminution indicator, with larger median particles suggesting poorer breakdown of the test food. Mean particle size may provide a complementary summary but could be more sensitive to unusually large or poorly segmented fragments. Maximum particle size may be useful as a descriptive indicator of incomplete comminution, while particle count may provide supporting information about the extent of food breakdown. However, these interpretations remain provisional until the outputs are validated against established chewing-function assessments and clinically meaningful thresholds are identified.

Our current evaluation quantifies algorithmic accuracy against known values in synthetic images rather than validating the measurement of the target construct (chewing ability), highlighting a key gap to fill. No established error threshold exists for image-based comminution or related chewing assessments. Performance is typically judged relative to a reference test and its clinical interpretability, rather than a fixed %-error rule. Accordingly, a suitable next step would be to evaluate the utility of the algorithm's outputs (particle count and size) against those from established tasks, including (i) the Masticatory Normative Indicator (4) and (ii) two-colour gum mixing (12, 26). Agreement metrics and clinical interpretation will provide a practical, clinically oriented assessment of the usefulness of the algorithm's outputs and possible suggestions for refinement.

### 4.3 Pathway From semi-automation to full automation

The GUI-based workflow introduces a semi-automated stage in which users visually inspect the calibration and segmentation steps and adjust key parameters to obtain the most appropriate segmentation for a given image. Although this introduces a highly subjective element, it may potentially serve an important developmental role: each manually optimised parameter set captures how visual inspection compensates for lighting, reflections, occlusion, noise, and other image-specific artefacts. With sufficient data, these human-adjusted examples could be used to train adaptive models that learn to propose appropriate parameter values automatically. This offers a practical pathway toward full automation, in which future machine-learning approaches could infer optimal calibration and segmentation parameters directly from image characteristics, reducing user burden and increasing consistency across images. As such, the current semi-automated system provides both a functional interim solution and a foundation for future fully automated processing.

Related work in oral function and swallowing assessment suggests that AI-driven approaches offer a feasible route to improved future remote assessment. Recent systems have used machine and deep learning, along with wearable or smartphone-based sensors, to evaluate swallowing ability, dysphagia risk, and chewing function from multimodal data (10, 27–29). Convolutional neural network (CNN) architectures such as VGG-16 can also be fine-tuned to categorise particle contours without retraining entire networks from scratch (30). Building on the current semi-automated pipeline, future work could therefore explore CNN-based contour recognition and learned parameter selection to reduce dependence on manual tuning while preserving transparency in how particle areas are estimated.

### 4.4 Limitations

#### 4.4.1 Image capture and calibration

Slanted light posed a problem when calibrating some images, producing shadows below the sample (see Additional File A3 for examples). At present, this can only be managed by manually adjusting the saturation and brightness cut-off values. The current mat design also places the Petri dish close to the curved black corners, so the outline around the dish can be partly occluded, which impacts calibration. Future iterations could increase the distance between the dish and the grid corners or add a second, larger outlining frame so that all four corners and the scale marker remain fully visible. For similar reasons, writing near the samples or calibration contours should be avoided. The current evaluation also used a limited range of image-capture devices and conditions, including one smartphone model during feasibility testing. Future work should assess robustness across different smartphones, camera settings, lighting conditions, and home environments.

#### 4.4.2 Parameter sensitivity, subjectivity and user impact

As discussed, algorithm performance is sensitive to internal configuration parameters, including saturation and brightness thresholds, erosion and dilation, and the minimum distance between segments in the watershed step. Small changes in these values can alter the number and size of particles detected, particularly in suboptimal images. The semi-automated GUI allows users to compensate for this by tuning parameters with visual feedback, but this introduces a subjective element, since users decide what is acceptable, and it becomes onerous when large numbers of images need to be manually and individually processed. Overlapping or clumped particles remain a particular challenge, as these can affect segmentation accuracy and may require manual or semi-manual adjustment. Usability may also vary across users, particularly older adults or people with reduced dexterity, and further work is needed to determine whether participants can independently prepare samples and capture usable images in home settings.

At this proof-of-concept stage, parameter tuning was undertaken by a single expert user, and inter-rater or intra-rater consistency was not assessed. Future work should evaluate whether different users arrive at comparable parameter settings and segmentation outputs. The GUI could also be used with image-analysis specialists and subject experts to define refined default parameters, acceptable-segmentation criteria, and decision rules for when manual adjustment is required.

#### 4.4.3 Measurement validity and clinical interpretation

The present work focuses on analytical accuracy for particle count and area, rather than on clinical validity or utility. The metrics have not yet been compared systematically with established chewing assessments, nor linked to clinically meaningful thresholds. Further studies are needed to validate the outputs against reference tests and to determine how they should be interpreted and used in clinical decision-making, in line with existing recommendations for evaluating masticatory-performance methods (3, 17). Furthermore, a key limitation of this work is that validation was conducted using generated geometric shapes and synthetic particle images, rather than real masticated carrot samples with reference measurements. Future work should validate the algorithm against expert annotation, flatbed scanning, wet sieving, or other established chewing-function assessment methods.

## 5 Conclusions

This paper reports the development and initial analytical evaluation of an image-based algorithm designed to support future remote measurement of chewing function. Our findings demonstrate the analytical feasibility of estimating comminution-related particle-size metrics from smartphone images and highlight current sources of error (for example occlusion of perimeters, overlap, calibration, and lighting sensitivity). In its current form, the method achieves its best performance through a semi-automated workflow, in which users adjust calibration and segmentation parameters via a GUI and rely on visual inspection to judge acceptable outputs. This introduces a subjective element and limits scalability, but provides a practical way to handle heterogeneous image conditions and a foundation for developing more adaptive, fully automated versions of the algorithm.

By validating performance against established reference tests and ensuring usability across diverse users and settings, the approach could form part of a future scalable toolkit for monitoring chewing ability remotely. Short-term priorities include protocol refinements to improve standardisation of image capture and calibration, reduction of manual parameter tuning, and prospective clinical validation against established methods. If validated against established reference tests and shown to be usable across diverse home settings, future versions of this approach could contribute to low-burden, longitudinal monitoring of chewing function, particularly in populations for whom clinic visits are difficult.

## Data Availability

The code and data presented in this study can be found in online repositories. The names of the repository/repositories and accession number(s) can be found below: Open Science Framework repository, https://osf.io/kgx9f.
