# Supplementary material for: A novel image-based algorithm to support future remote assessment of chewing function
Source: Front Dent Med. 2026 Sep 3;7:1870425. doi: 10.3389/fdmed.2026.1870425 (PMC13581933; doi:10.3389/fdmed.2026.1870425)

Additional Files

Additional File A1.pdf -

Plastic lightbox used for standardising image capture

Image of the lightbox that was used during the lab trials to standardise image capture.

Additional File A2.pdf

Expanded workflow diagram illustrating calibration and determination processes

Diagram detailing all calibration and determination processes involved in the algorithm development.

Additional File A3.pdf

Chewed carrot samples with shadows.

Example image of chewed carrot samples demonstrating shadows caused from tilted light sources.

Additional File A4.pdf

A User's Guide for the Image-Based Chewing Estimation Algorithm

Written instructions for using the RIICE algorithm.

Additional File A1. *Plastic lightbox used for standardising image capture*

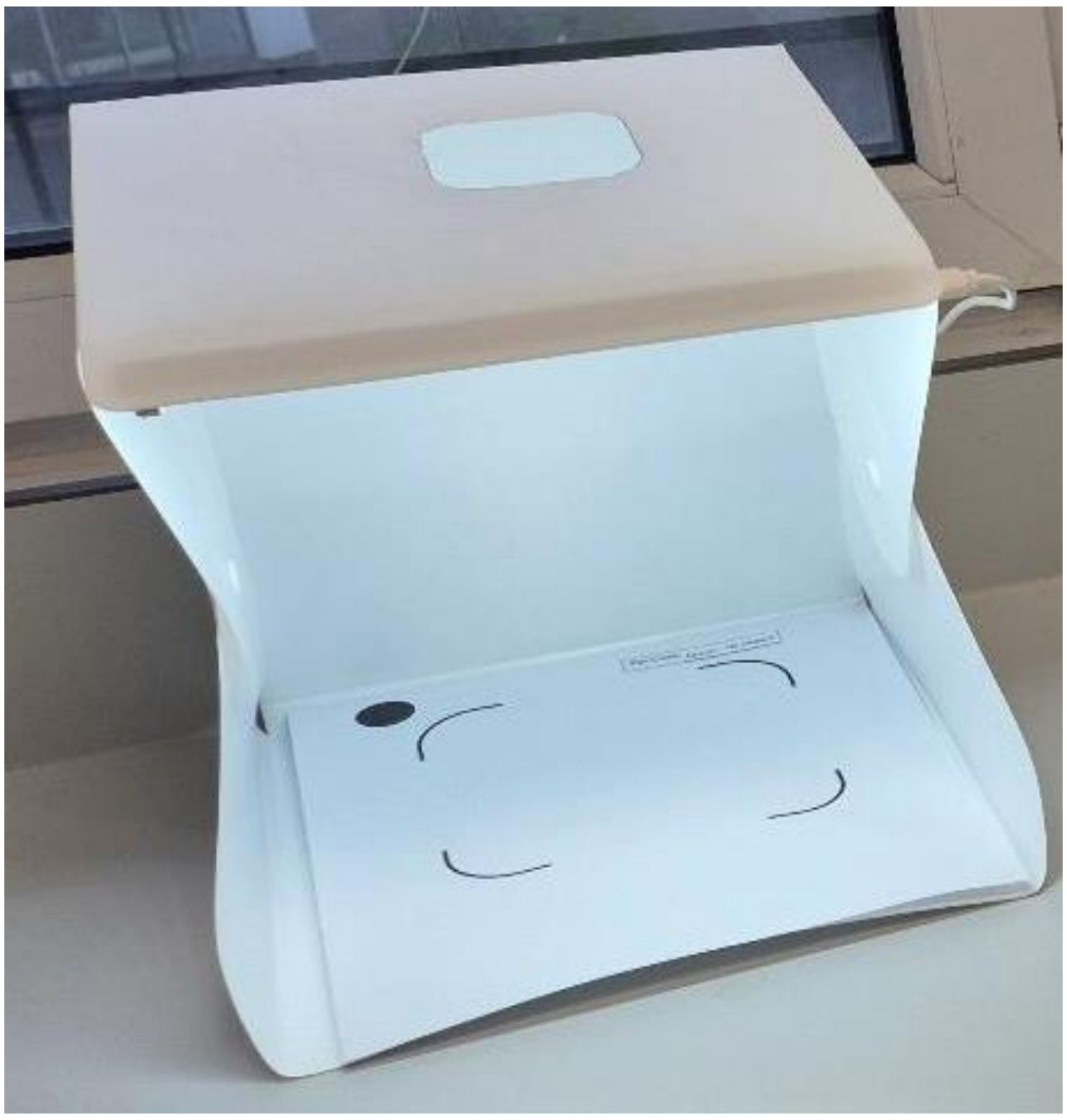

Additional File A2. *Expanded workflow diagram illustrating calibration and determination processes.*

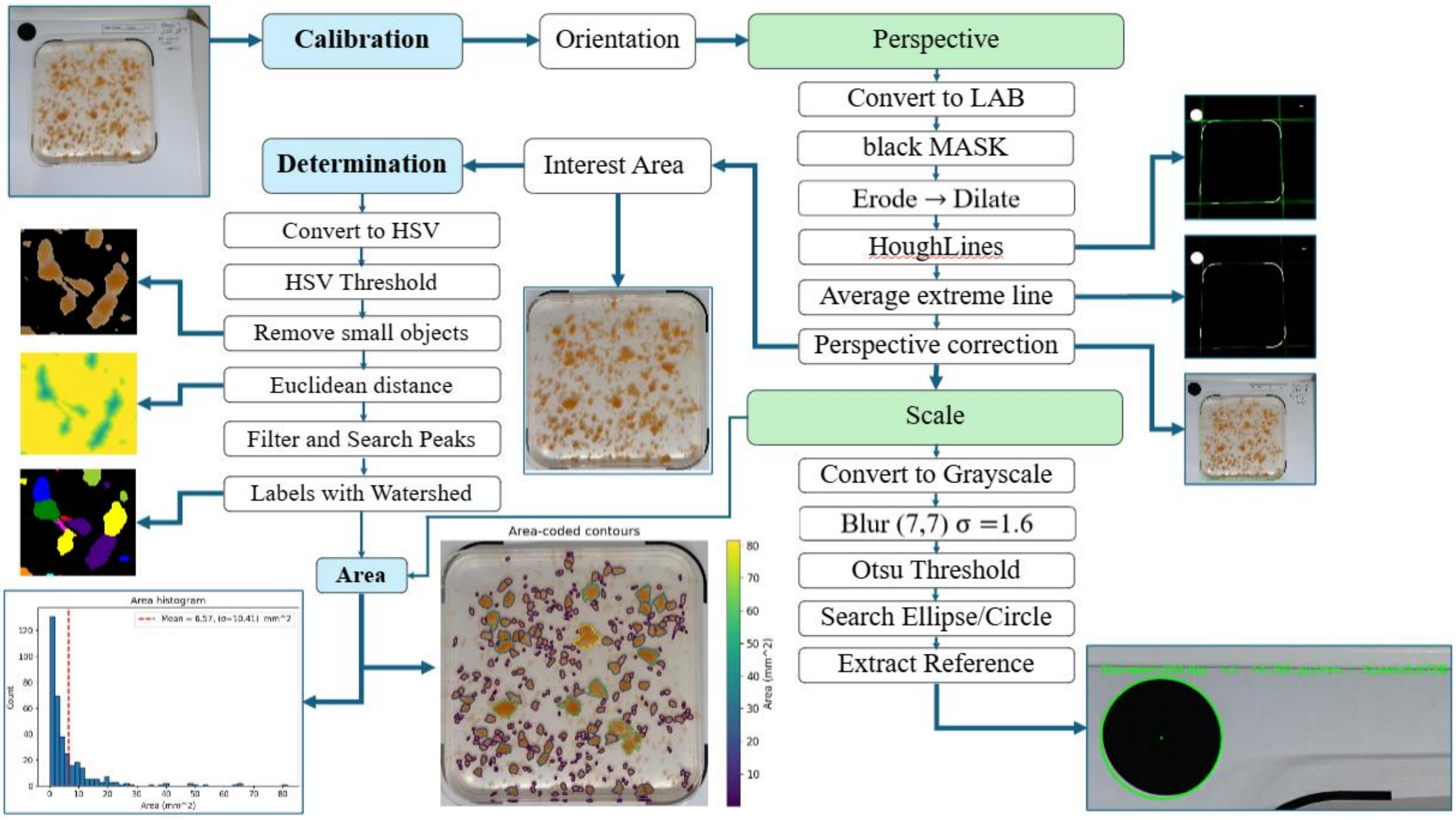

Additional File A3. *Chewed carrot samples with shadows caused by tilted light sources.*

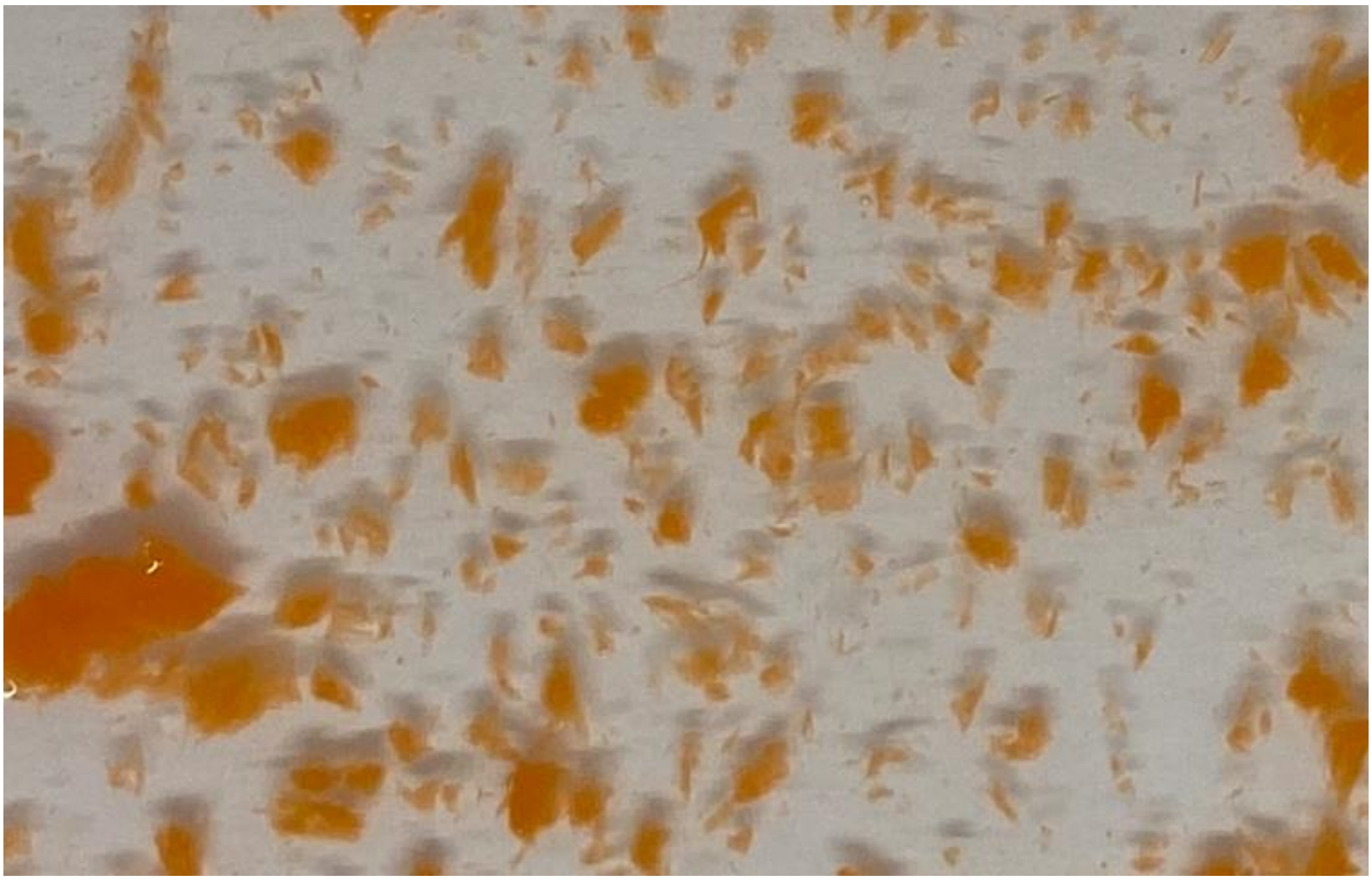

Additional File A4. *A User's Guide for the Image-Based Chewing Estimation Algorithm*

riiceproject.uk

RIICE: Remote Intervention to Improve Chewing Efficiency

A User's Guide for the Image-Based Chewing Estimation Algorithm

Availability of data and code: Python scripts, Graphical User Interface (GUI) files, sample images, and related documents are available on the Open Science Framework (OSF; osf.io/kgx9f).

The image-based chewing estimation algorithm can currently be used in two ways: Option A is a Python script, which runs the full pipeline and allows calibration settings and image parameters to be edited directly; Option B is a graphical user interface (GUI), which provides an interactive, semi-automated workflow for users who prefer visual feedback and parameter adjustment. The Python scripts use a set of recommended parameters, but also allow users with Python coding skills to edit these parameters in the script. The GUI, by contrast, requires no formal coding skills: it allows users to load individual images, visualise each stage of the processing pipeline, and interactively adjust key parameters via sliders and checkboxes. Adjustment of image parameters supports more accurate particle segmentation and area estimation when imaging conditions deviate from the ideal (for example due to variable lighting, reflections, or other image capture noise), and provides an accessible way to use the chewing estimation algorithm.

Option A. Implementing the Script in Python

1. Read the *README.md* file and follow the instructions to prepare the code and install the required Python packages.
2. From a terminal in the project directory, execute: *python main.py*. A file selection window will open for you to choose an image, after which the script will run the full processing pipeline and display the outputs.

By default, the script uses a recommended set of image processing parameters that work well under relatively consistent imaging conditions. Users with experience in Python can also modify these parameters directly in the script (for example the HSV thresholds, and image calibration settings) to adapt the algorithm to different datasets or to run batch analyses. However, this option generally

requires familiarity with editing and running Python code, and does not provide the same visual, interactive feedback as the recommend GUI (see below).

Option B. Implementing the Graphical User Interface (GUI)

1. Read the *README.md* file and follow the instructions to prepare the code and install the required packages.
2. From a terminal in the project directory, execute: *python gui_main.py*. The GUI will open and no further coding is required.

The GUI provides an interactive, semi-automated workflow. Users can load individual images, inspect each stage of the processing pipeline, and adjust parameters via sliders and checkboxes. The following is a sample workflow of how to use the GUI and examples of adjustments to image parameters.

Step 1. Load target image

The file selection button (highlighted in red in Figure 1 below) is used to open and load an image file (e.g. JPEG or PNG). This view allows the user to confirm that the correct image has been loaded and to inspect its general quality (lighting, reflections, visibility of the reference disc, etc.).

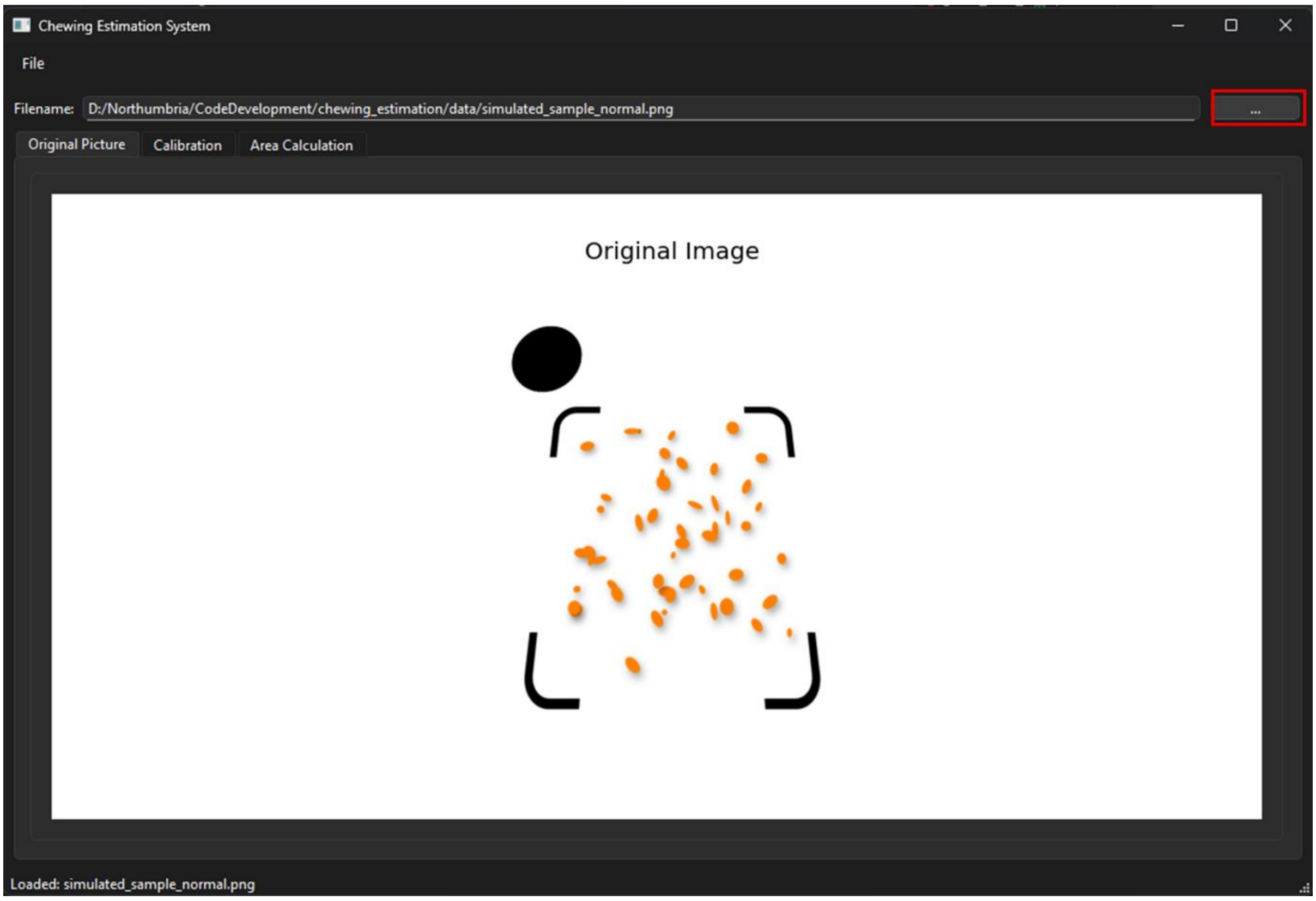

Figure 1. Loading target image

Step 2. Calibrate image settings

The 'Calibration' tab (see Figure 2 below) is used to detect the container and reference disc, isolate the target region of the image, and estimate the pixel-to-millimetre scale.

- Erode / Dilate: The user can select the number of erosion and dilation iterations. Erosion helps remove small noisy artefacts, while dilation can reconnect broken edges or slightly enlarge structures.
- Minimum and maximum line gap: Adjusting these sliders changes how lines are detected and connected, helping the algorithm correctly identify the container and reference disc.
- Remove text: The 'Remove text' option is an auxiliary filter to remove fine lines or printed text that might interfere with line detection.
- Reference scale dimension: The user specifies the physical size (in mm) of the circular reference disc. This is used to compute the scale factor (pixels per mm).

The calibration plots provide visual feedback on whether the container and reference disc have been correctly detected.

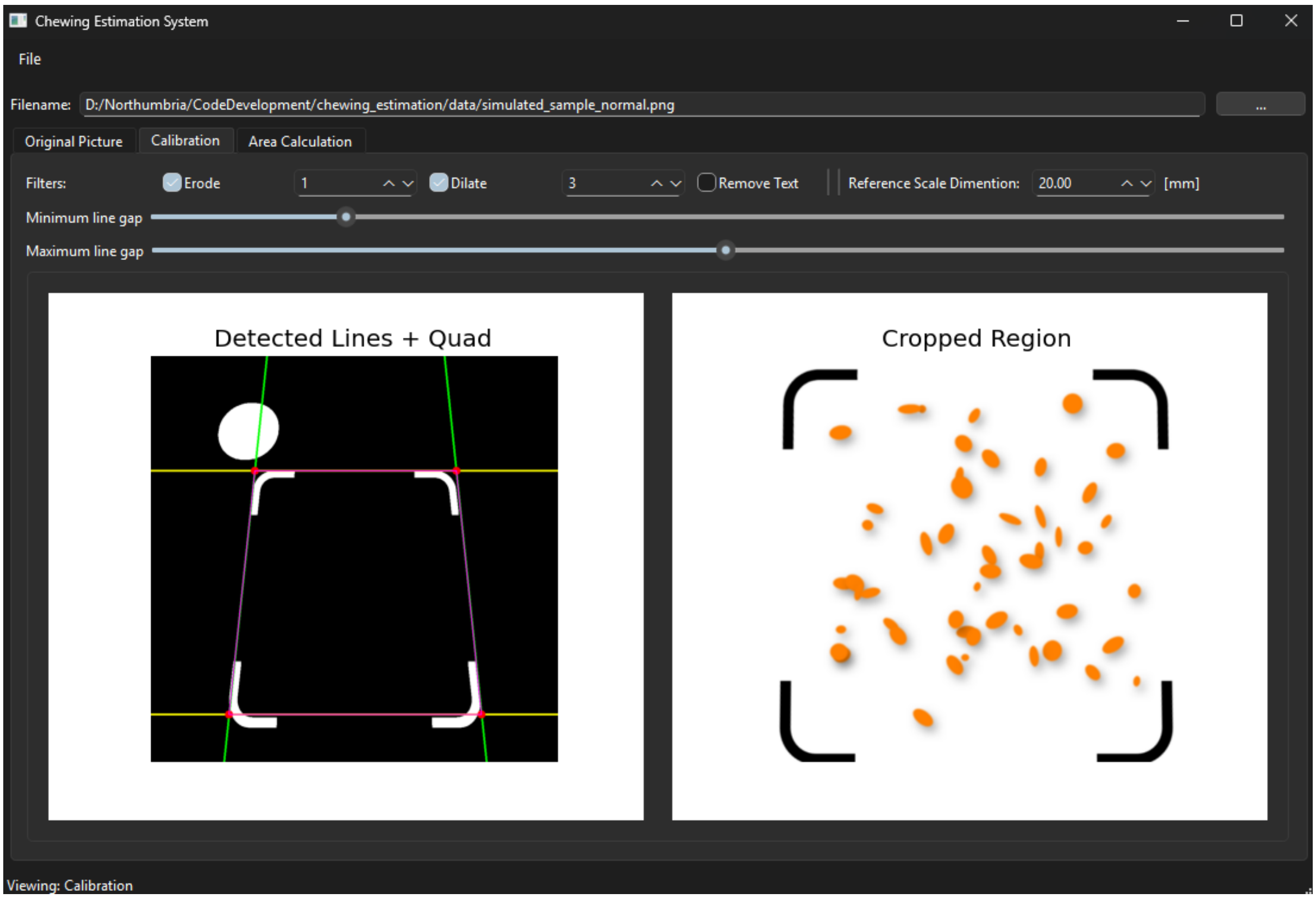

Figure 2. Calibrating image settings

Step 3. Calculate particle areas

In the 'Area Calculation' tab (see Figure 3 below), the user adjusts settings used to detect and measure the chewed particles. When the default parameters under-detect particles that should be included, or over-detect small fragments and noise, these settings can be tuned so that the segmentation more closely matches the intended set of chewed particles. This introduces an inherently subjective element, as users rely on visual inspection to judge whether the segmentation is appropriate, and reflects the semi-automated nature of the current approach. Visual feedback is provided by adjusting the following parameters:

- Saturation and brightness cut-offs: Use the saturation and brightness sliders to define which pixels belong to the particles and which belong to the background.
- Area view (direct vs ellipse): Two visualisation modes are available:
    - Direct Area View shows the segmented contours mapping directly onto the image particles.
    - Ellipse Area fits an ellipse to each particle and reports the area of the fitted ellipse.

  Switching between views helps assess whether particles are being segmented appropriately.
- Minimum sample distance: The Minimum Sample Distance (px) setting controls the minimum spacing between particle centres used when separating adjacent particles. Increasing this value can help split merged particles; decreasing it can reduce over-segmentation.

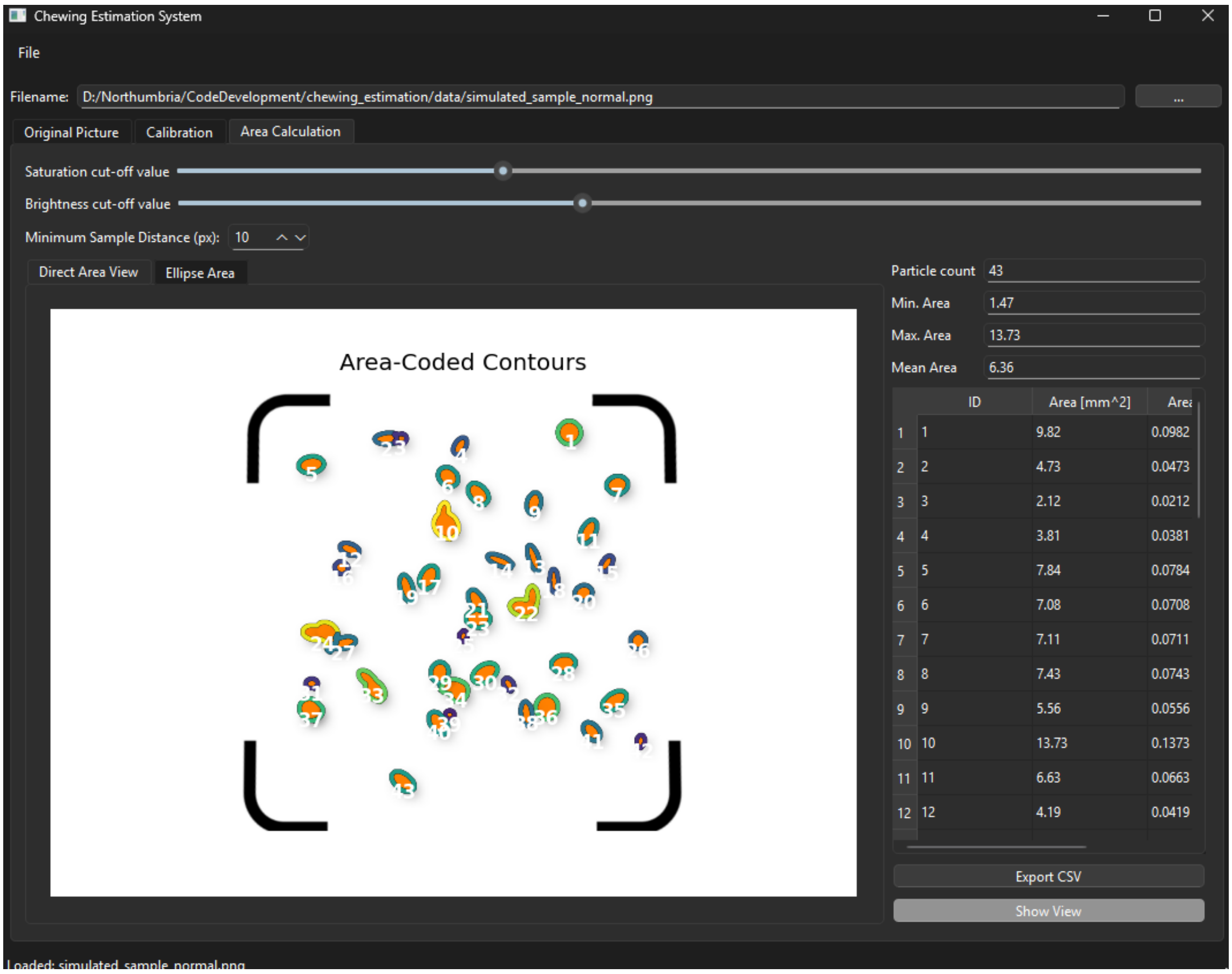

Figure 3. Calculating particle areas

Step 4. Review and Export results

After visually inspecting the results and adjusting the parameters as needed, the user can then review and export the quantitative outputs.

- The GUI reports the particle count, as well as the minimum, maximum and mean areas in the metrics panel (highlighted in red in Figure 4).
- A table lists each particle ID with its area in mm^2 and cm^2.
- Results can be exported as a CSV file for further analysis using the 'Export CSV' button (also highlighted in Figure 4 below).

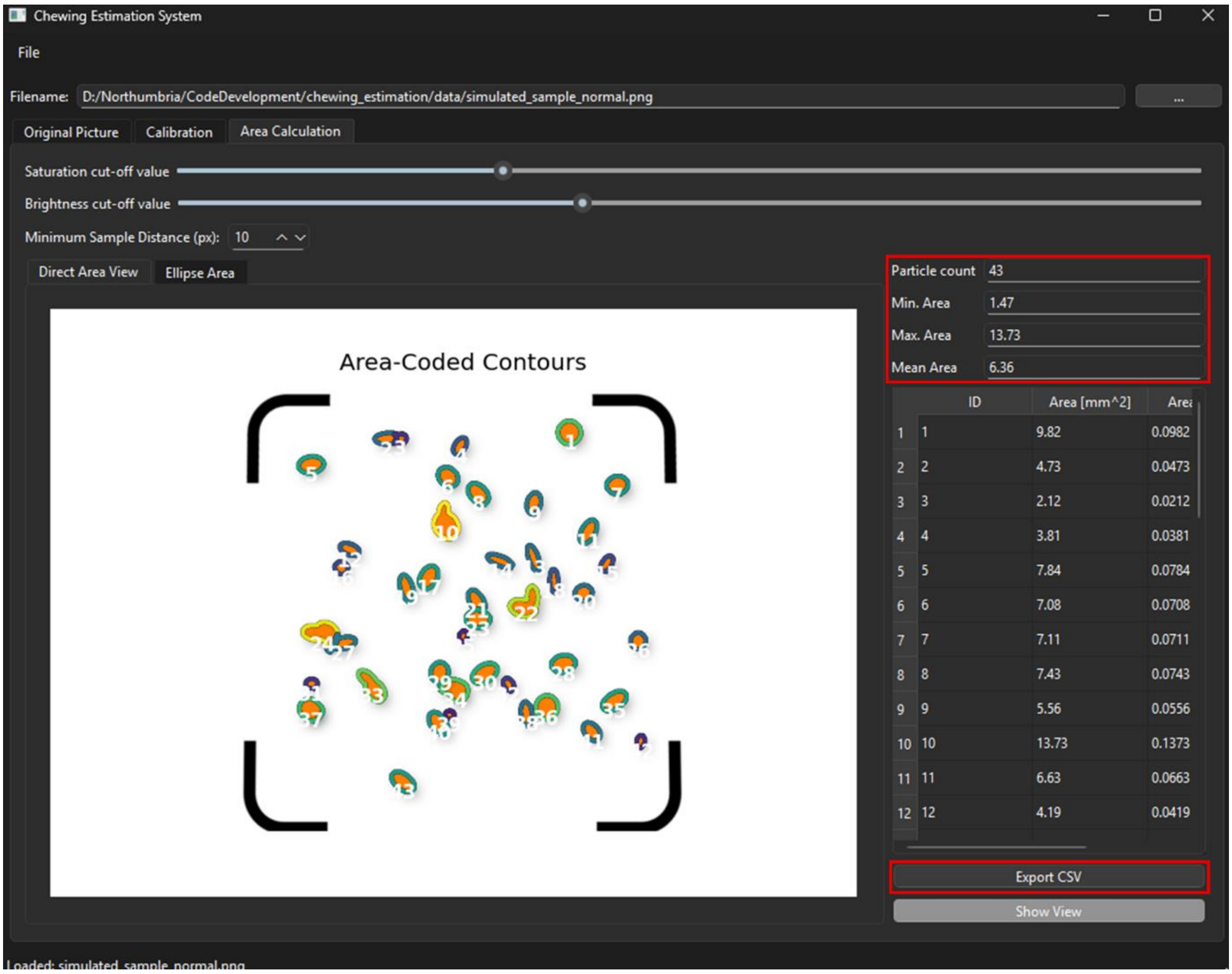

Figure 4. Reviewing and Exporting Image Metrics

Worked Examples of GUI Parameter Adjustment

To illustrate how the GUI can improve segmentation in practice, we include a set of worked examples for both the Calibration and Area Calculation tabs. For each example, the left-hand panel shows the output obtained using the default parameter settings, and the right-hand panel shows the result after manual adjustment of the relevant parameters (for example erosion and dilation, line gaps, saturation and brightness cut offs, and minimum sample distance). These side-by-side comparisons highlight typical issues such as missed particles, merged particles or background noise, and demonstrate how visual inspection and parameter tuning can produce a segmentation that more closely represents the displayed image.

Calibrating Image Settings

Several features of the sample images affected successful calibration. These included the visibility of the grid corner markings, occlusion of markers from the Petri dish, and the presence of text or drawn lines that were sometimes mistakenly identified as grid markings. There were also occasional failures to detect the reference circle and grid lines. As shown in the worked examples (see Table A1 below), this often led the algorithm to select an incorrect region of interest. In most cases, however, adjusting the calibration parameters allowed an acceptable fit to the target image area to be achieved.

It should be noted that, during testing, the algorithm failed to calibrate several images even when they were visually similar to successfully calibrated examples and despite attempts to adjust relevant settings. This likely reflects a combination of suboptimal image conditions and current limitations in the algorithm's flexibility. These issues point to the need for improved image capture procedures, as well as future refinement and further development of the calibration stage to increase algorithm robustness.

Table A1. Default and user-adjusted calibration of sample images

	Calibration with default settings	Calibration with user-adjusted settings

<table>
<tr>
<td>Sample
Image A</td>
<td>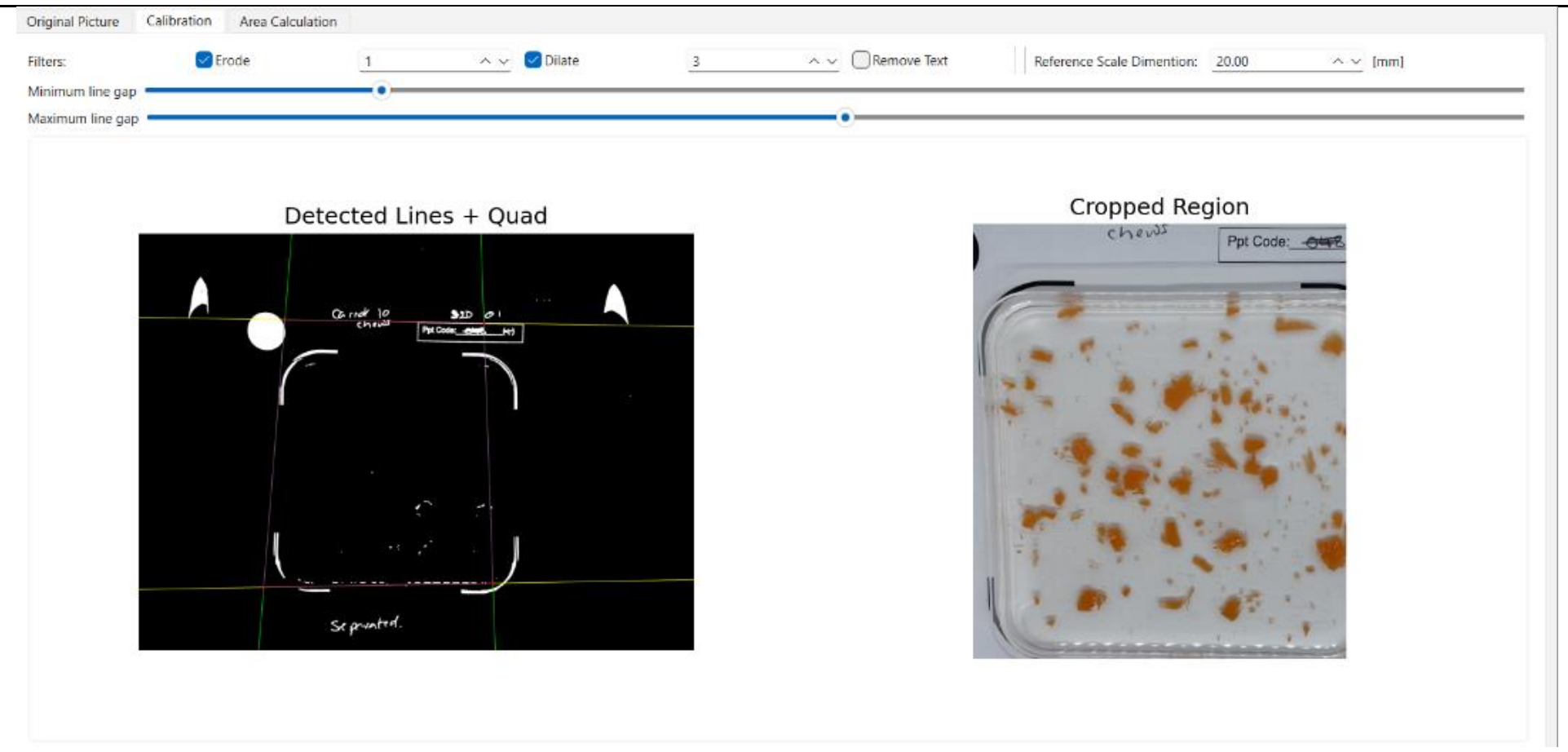
</td>
<td>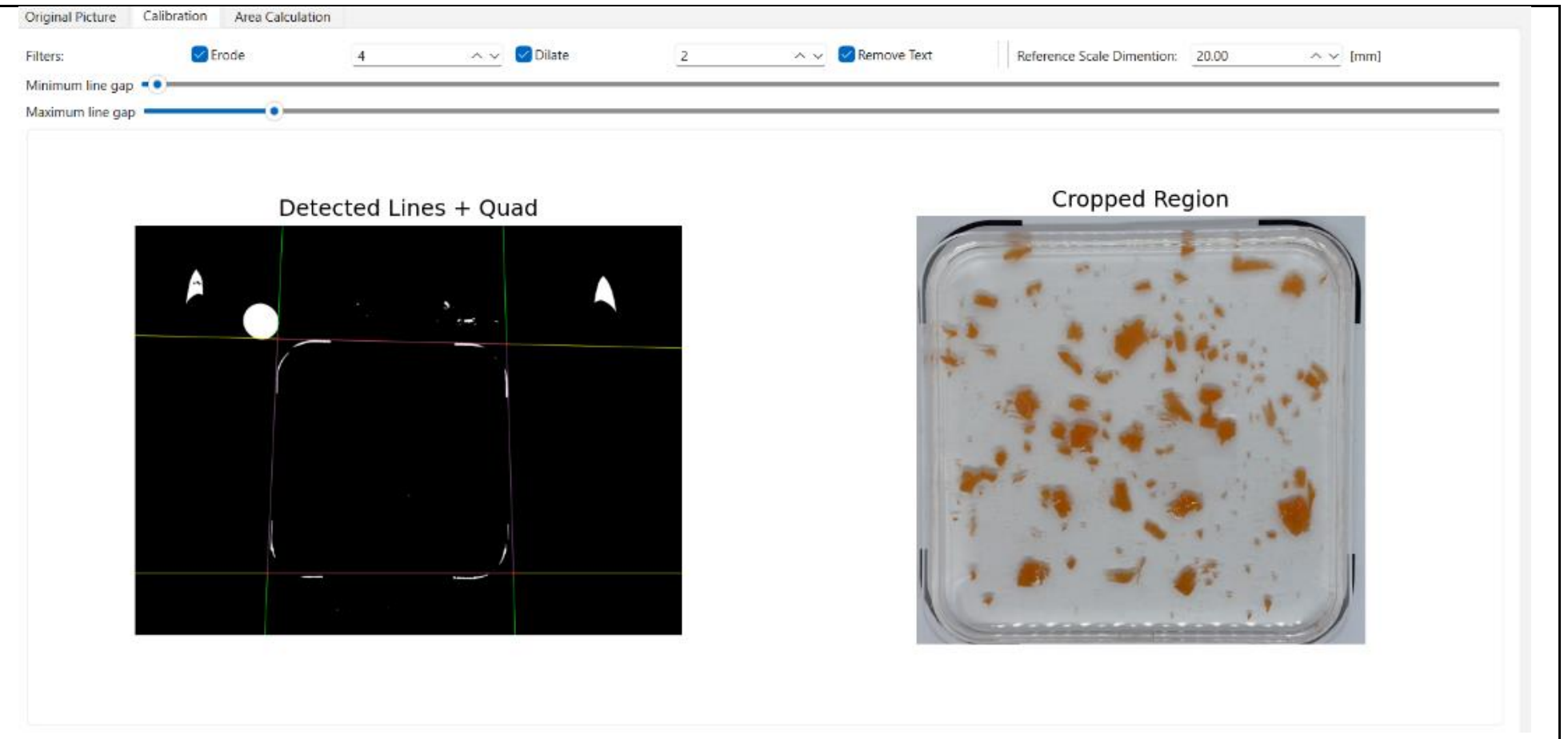
</td>
</tr>
<tr>
<td>Sample
Image B</td>
<td>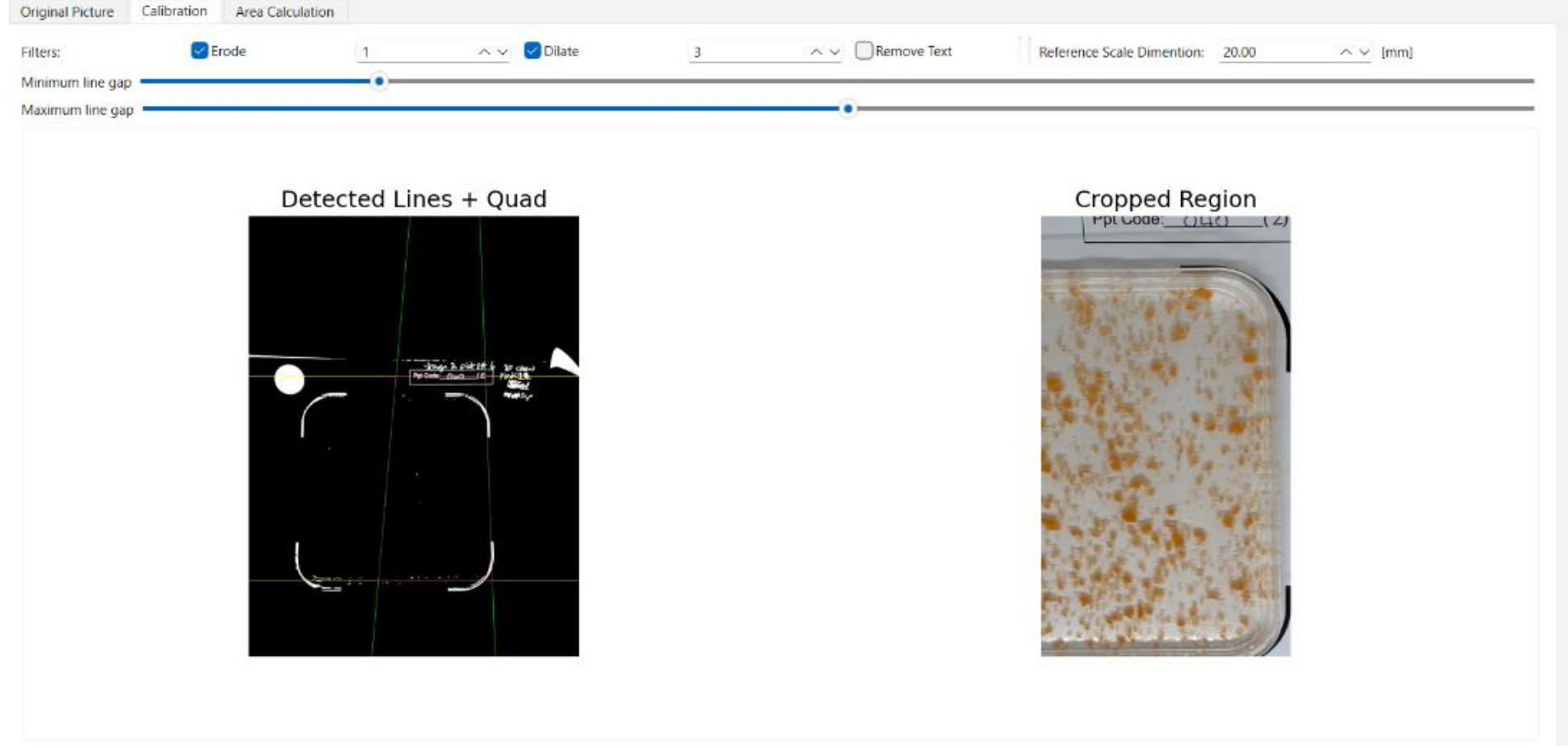
</td>
<td>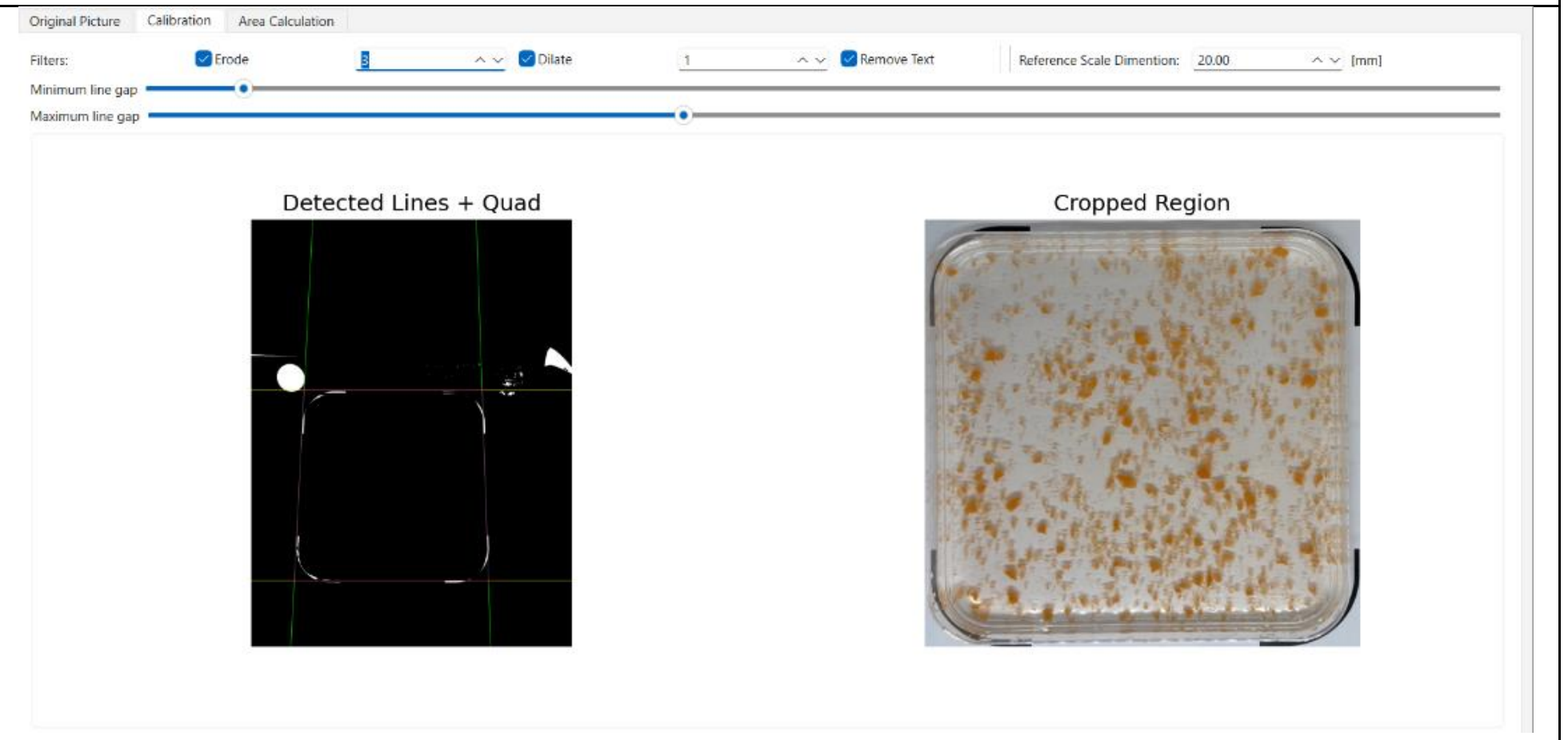
</td>
</tr>
</table>

Sample Image C	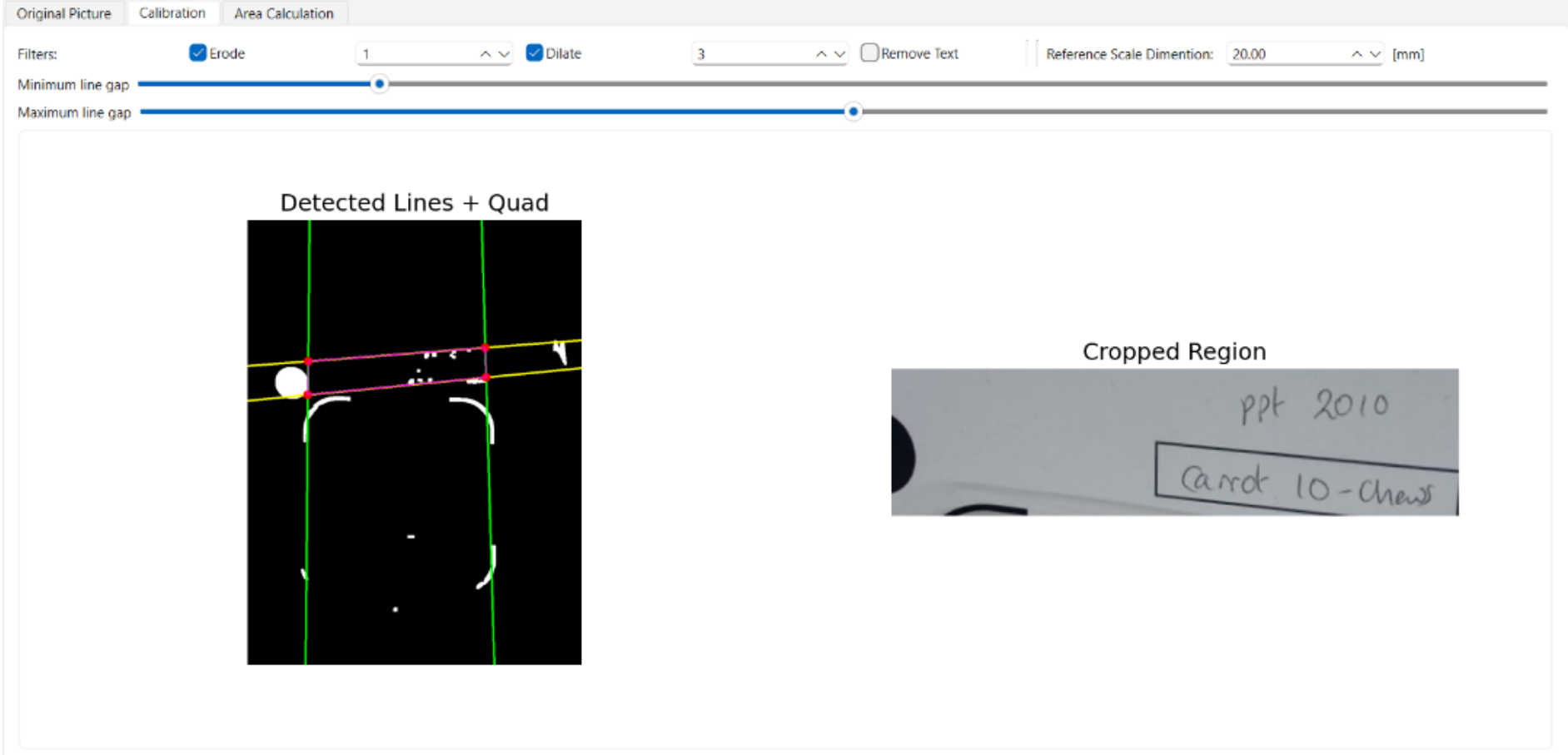	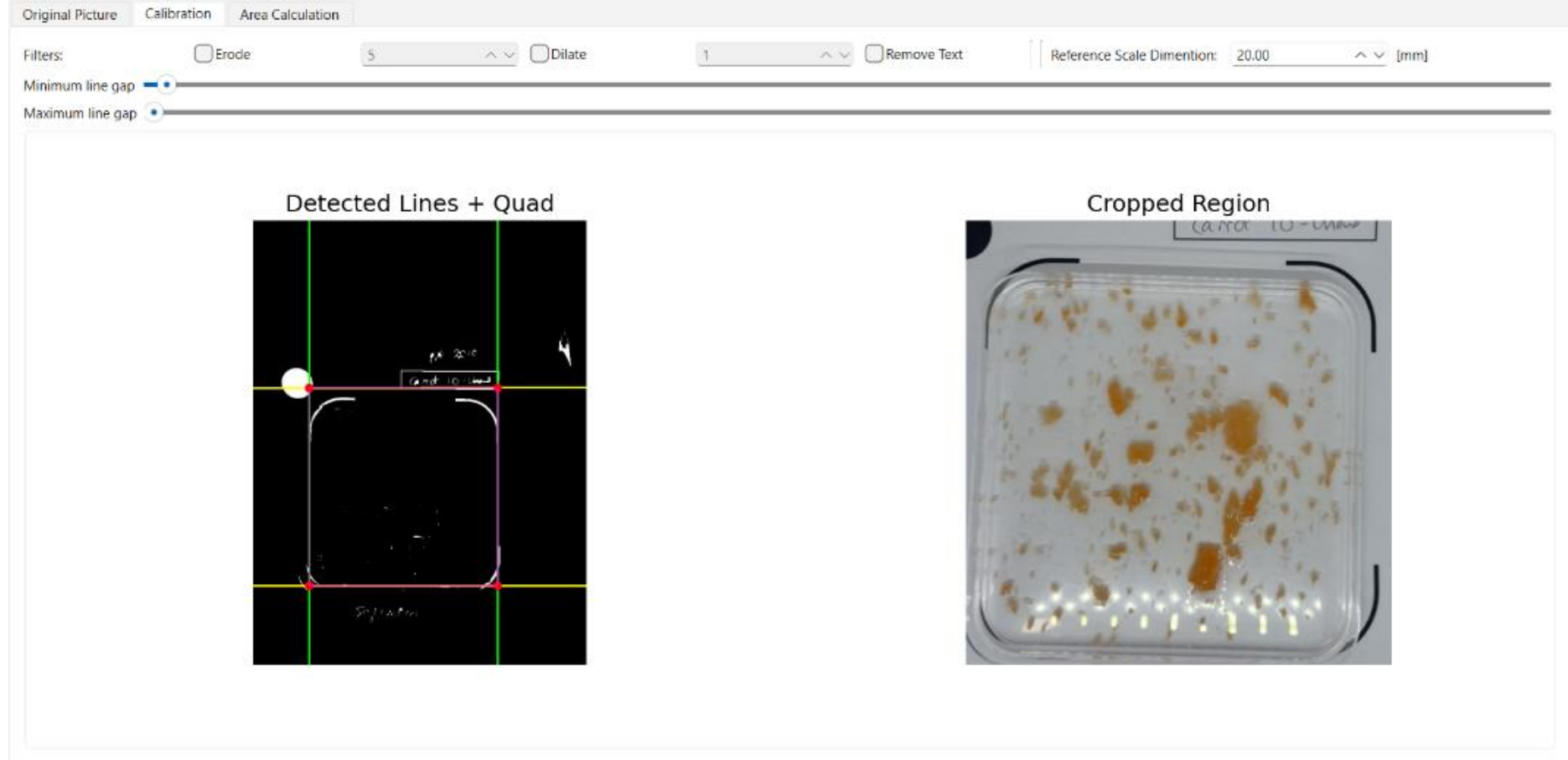

Calculating Particle Areas

For particle counts and area calculations, the brightness and saturation controls allow users to adjust the thresholds used to segment and include target particles. As shown in the worked examples (see Table A2), these adjustments directly influence the algorithm's output metrics. Features of the images can still lead to incorrect segmentation, for example reflections from the dish being counted as particles (seen as lines of bright white dots near the bottom of each sample image), grid markings or other spurious image artefacts being included, or shadows and overlapping pieces preventing effective separation of particles. Despite the imperfect nature of the process, adjusting these parameters demonstrates the potential to tune the algorithm to more accurately capture particle counts and area estimates.

Table A2. Default and user-adjusted area calculations for sample images

	Particle area estimates using default settings	User-adjusted particle area estimates

Sample Image A

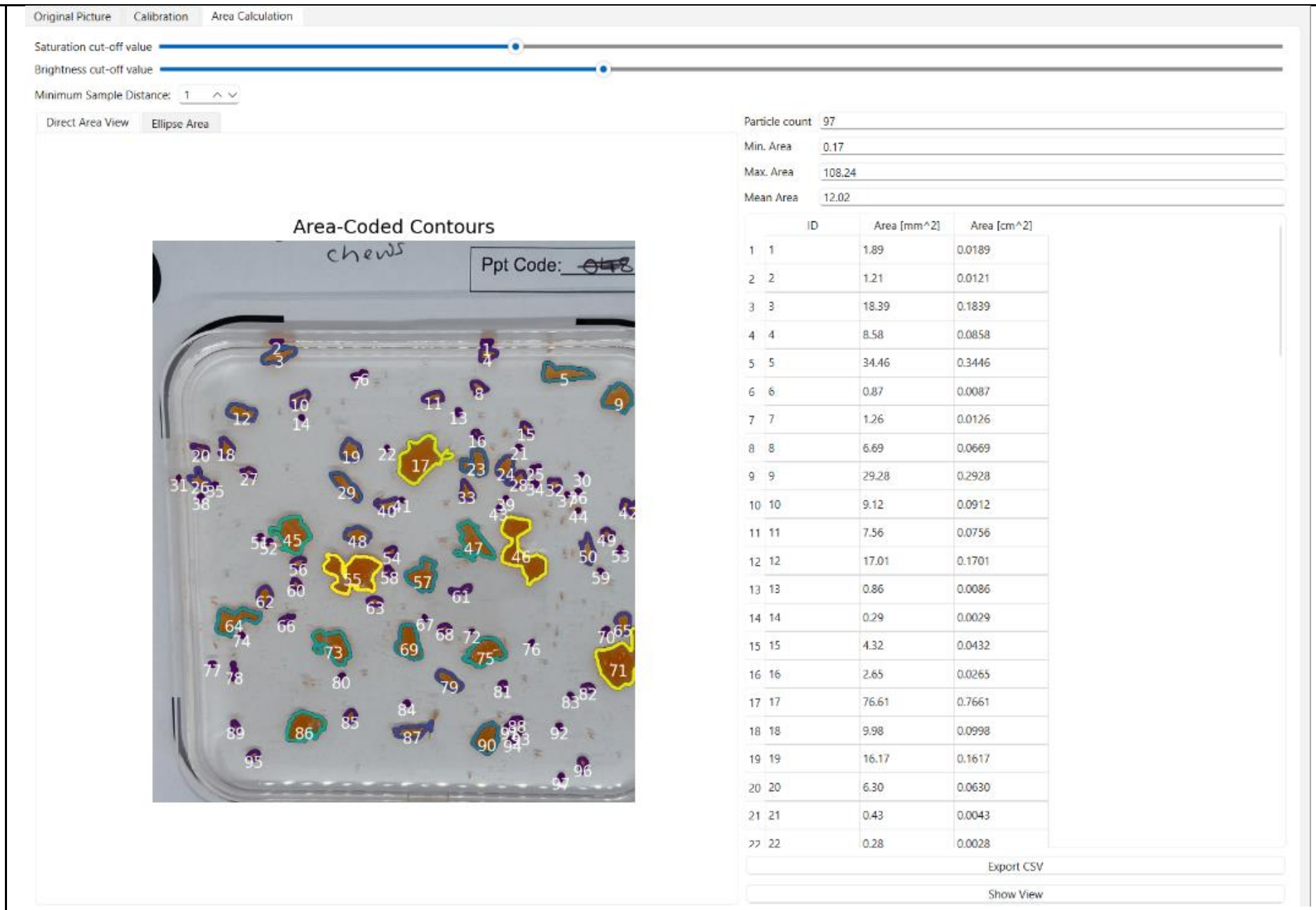

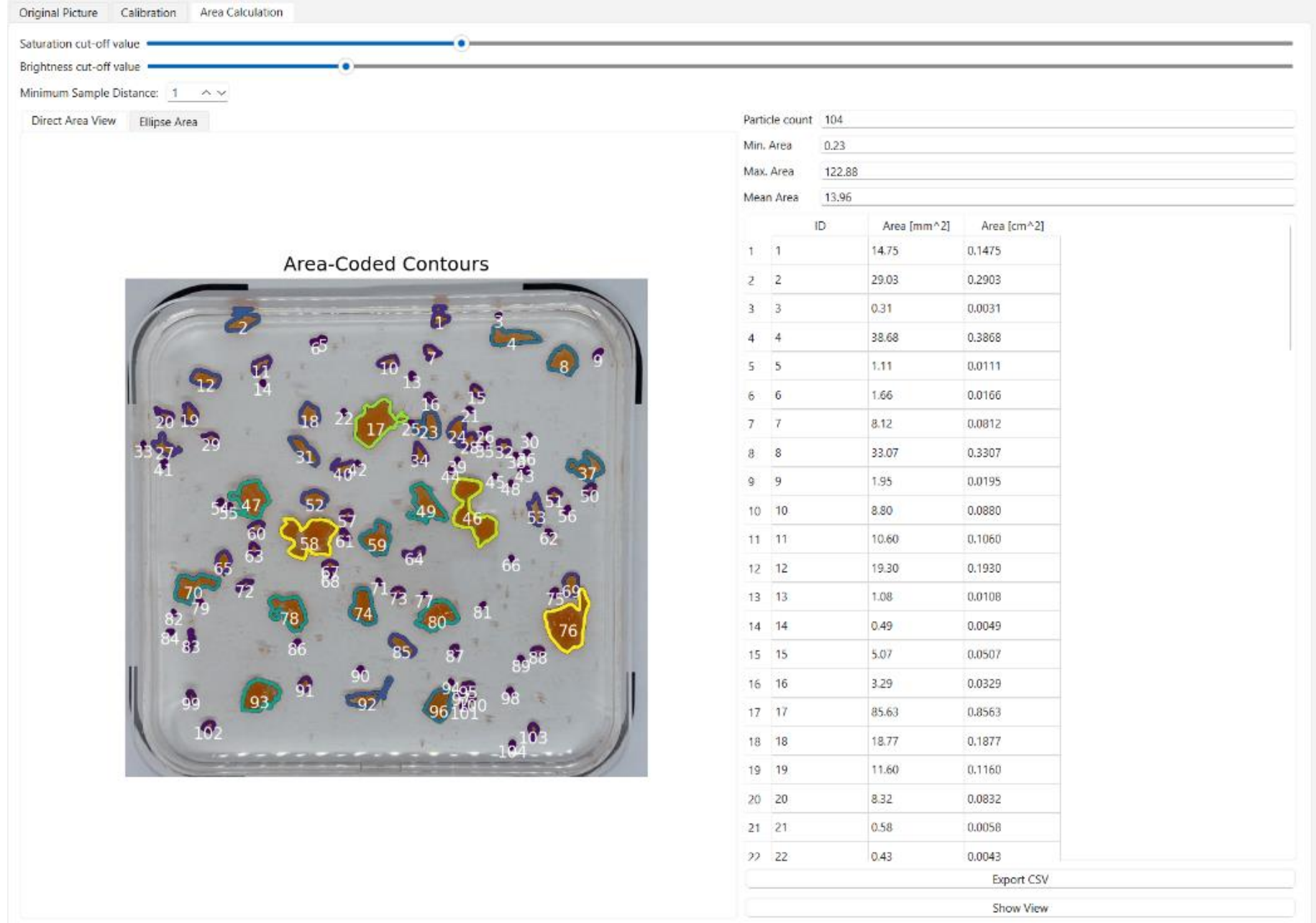

Sample Image B

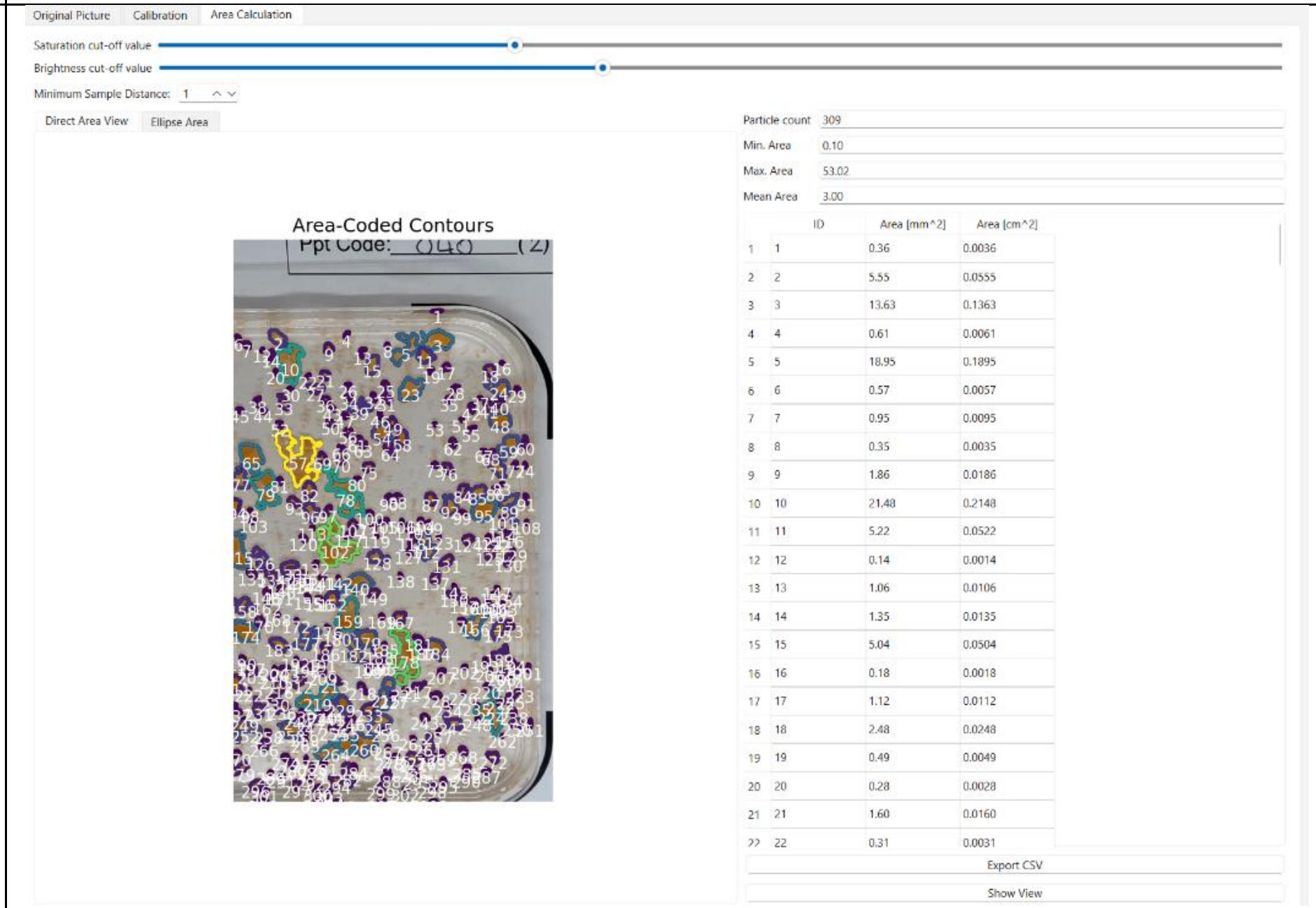

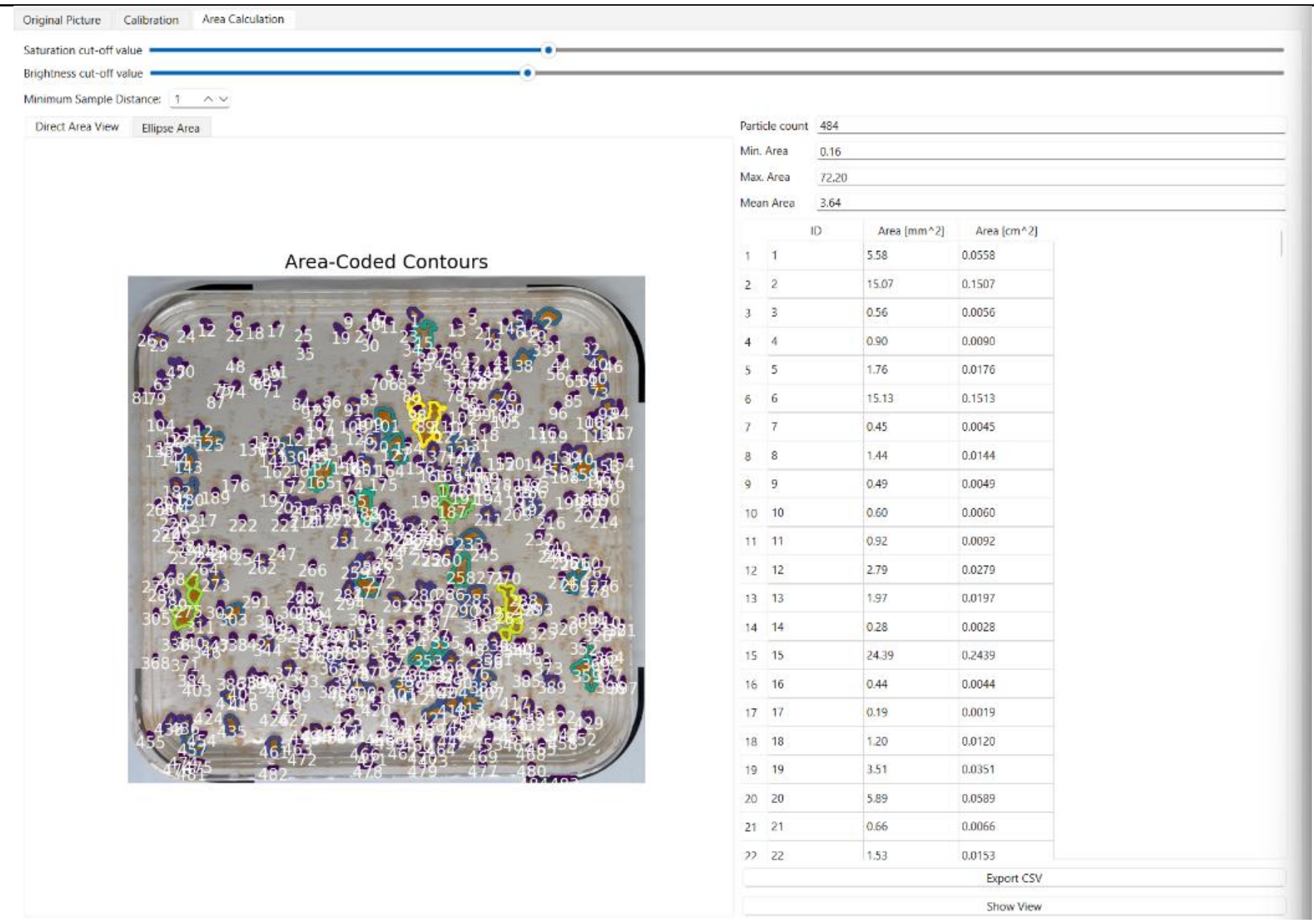

Sample

Image C

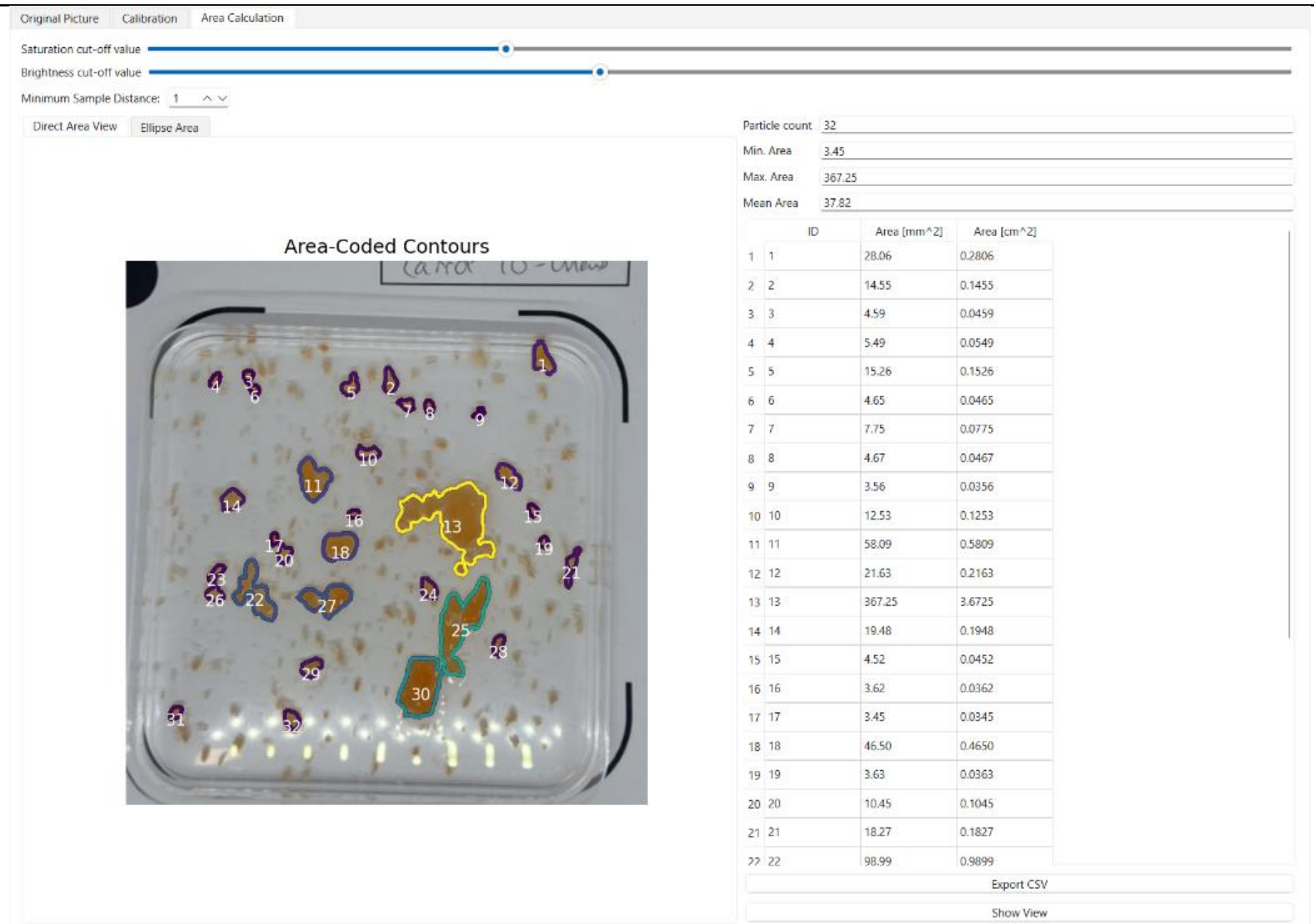

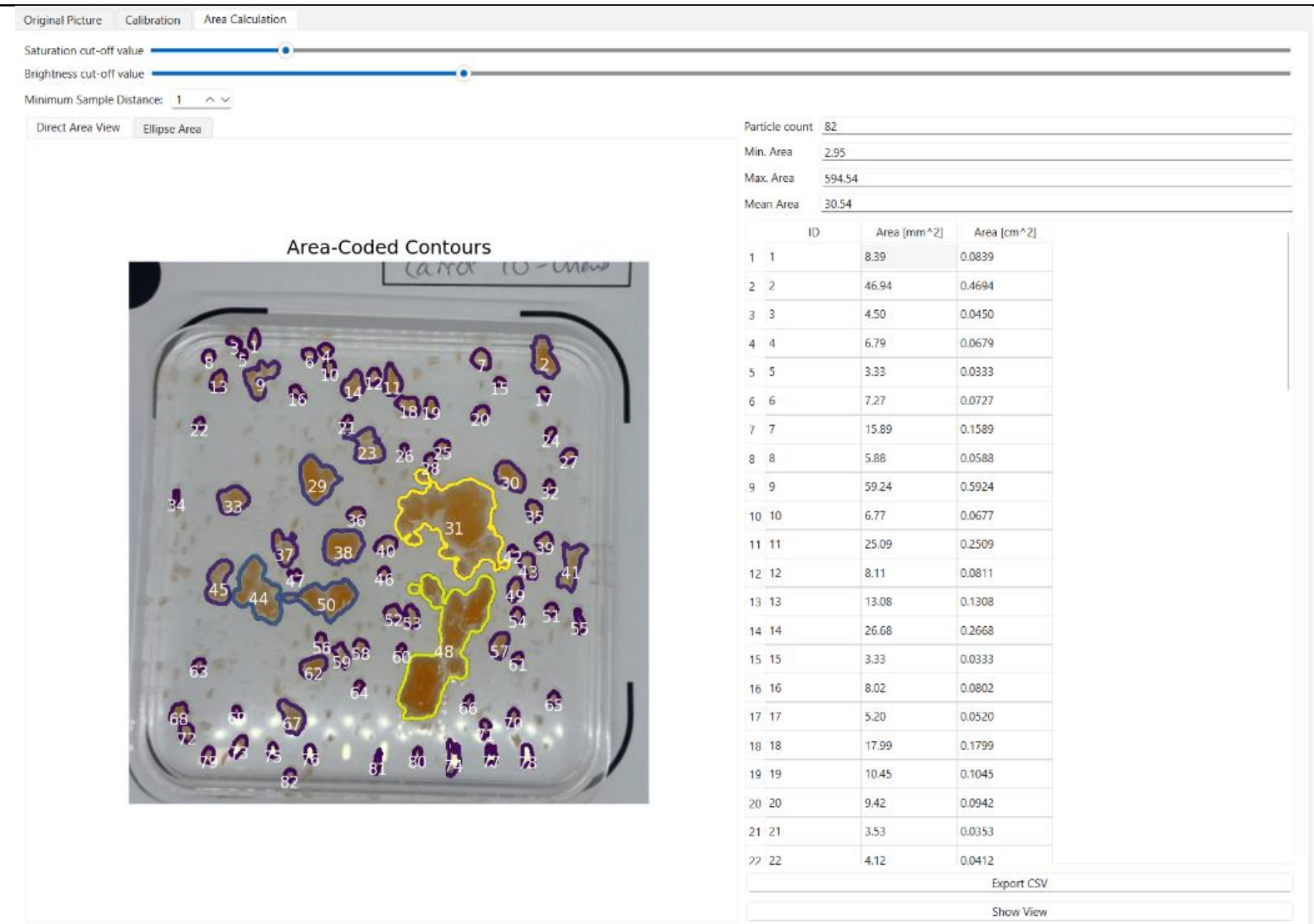

Supplement: Supplementary file 1 [file Datasheet1.pdf]
